# Quick and Green Microextraction of Pyrrolizidine Alkaloids from Infusions of Mallow, Calendula, and Hibiscus Flowers Using Ultrahigh-Performance Liquid Chromatography Coupled to Tandem Mass Spectrometry Analysis

**DOI:** 10.1021/acs.jafc.2c02186

**Published:** 2022-06-17

**Authors:** Natalia Casado, Begoña Fernández-Pintor, Sonia Morante-Zarcero, Isabel Sierra

**Affiliations:** Departamento de Tecnología Química y Ambiental, E.S.C.E.T., Universidad Rey Juan Carlos, C/ Tulipán s/n, 28933 Móstoles, Madrid, Spain

**Keywords:** pyrrolizidine alkaloids, edible flower infusions, microextraction, μSPEed, UHPLC−MS/MS, food safety

## Abstract

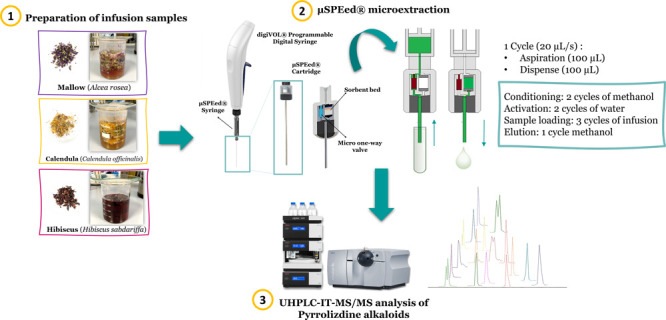

A sustainable microextraction of pyrrolizidine alkaloids (PAs) from edible flower infusions using the innovative μSPEed technique is proposed. Different sorbents and extraction conditions were tested, achieving the highest extraction efficiency with an octadecylsilane sorbent (4 mg). The extraction procedure just took 1 min per sample, and only 300 μL of methanol and 300 μL of the sample were used per extraction. Ultrahigh-performance liquid chromatography coupled to tandem mass spectrometry was used for analysis. The method was properly validated, providing suitable linearity, selectivity, sensitivity (quantification limits 0.3–1 μg/L), overall recoveries (79–97%), and precision (≤17% relative standard deviation). Its application to the analysis of different infusions of mallow, calendula, and hibiscus flowers revealed similar total PA values (23–41 μg/L) and contamination profile among the mallow and hibiscus samples, with predominance of senecionine-type and heliotrine-type PAs, respectively. Conversely, calendula samples showed more variations (23–113 μg/L), highlighting the occurrence of intermedine *N*-oxide and europine *N*-oxide on them.

## 1 Introduction

Plants belonging to the families Asteraceae, Fabaceae, Boraginaceae, Orchidaceae, and Apocynaceae are producers of natural toxins, so-called pyrrolizidine alkaloids (PAs), which can be found as potential contaminants in food.^1^ Some of these plants are consumed directly, such as borage, while other nonedible plants belonging to these families extensively grow as weeds in crop fields, often leading to the contamination of other food products. In this sense, it was first widely assumed that the contamination of non-PA-producing plants was due to the accidental inclusion of weeds or impurities from PA-producing plants during harvest or processing. However, in the last years, several works have demonstrated that besides cross-contamination during harvesting processes, other contamination paths are possible, such as natural horizontal transfer through soil, animal feed, food fraud, adulteration, and so on.^2^ The intake of these alkaloids is mainly associated to liver damage, but they can also produce genotoxic and carcinogenic effects at long-term exposure.^3−5^ Many food alerts have notified, in the last few years, high levels of these alkaloids in a wide variety of food products, making the occurrence of these toxins one of the main current problems in the food safety field.^2,6^ Particularly, 15% of these alerts have been indicated in teas and infusions made from plants and flowers (e.g., chamomile, spearmint, rooibos, nettle, and herbal mixes),^2,6^ as they are products that are increasingly consumed by the population for curative and dietary purposes.^7−10^

In this context, the intake of flower infusions, such as mallow, calendula, and hibiscus, is increasing due to their gastrointestinal, relaxing, anti-inflammatory, and expectorant properties, among others.^9,10^ However, there are no previous studies in the literature which analyze individual infusions of these flowers for the determination of PAs. Only a recent work from Kwon et al. performed the analysis of single dry hibiscus samples, among other dried herbal teas.^11^ In contrast, only a few works have reported the analysis of herbal mixed teas containing some of these flowers in their composition, but using this way, it is not possible to attribute the occurrence of PAs to a single flower.^12,13^

Another relevant issue is that many of the works published in the literature that determined PAs in teas or herbal teas, performed the analysis directly on the dry samples instead of the infusions.^11,14−23^ This is an important point to be considered, as some authors have confirmed that not always the transfer rate of PAs from the dry sample to the infusion is 100%.^12,24−26^ Therefore, it is more suitable to perform the analysis of infusion samples to achieve more reliable data of the real intake and the exposure of consumers to these alkaloids.

Accordingly, due to the potential risk for human health that the continuous and frequent intake of these products may entail, it is of utmost importance to monitor the occurrence of PAs in food by high-throughput analytical procedures. In this context, a regulation to monitor the occurrence of these alkaloids in some food products has recently been published, which includes maximum concentration levels for tea and herbal infusions in the range 75–400 μg/kg (for dried products) and 1.0 μg/kg (in liquid form) for teas and infusions intended for infants and young children.^27^

In addition, according to this legislation, every analytical methodology designed to control these contaminants in food must include a set of 21 PAs (including their *N*-oxides, PANOs). Likewise, 14 additional PAs, which are isomers of one or more of the previous 21 compounds and that are known to coelute with some of them, can also been considered if the chromatographic method employed for the analysis is able to separate and individually identified them without coelution problems.^27^

Given the large number of PAs to be monitored, for the determination of these compounds, it is necessary to perform a multicomponent extraction, and afterward, a multicomponent analysis, always considering the maximum concentration limits established for these alkaloids in their regulation.^27^ In this context, microextraction techniques have gradually gained attention in the last few years due to their many advantages over conventional extraction methods, such as the minimal use of organic solvents, the low amount of sample required, and the user-friendly systems, among others.^28,29^ Therefore, the use of these miniaturized techniques enables the development of environmentally friendly procedures, which meet the requirements of the Green Analytical Chemistry.

Among the wide variety of microextraction procedures with different formats and configurations, the μSPEed technique can be highlighted. μSPEed is a promising extraction technique, which is an improved variant of the microextraction by packed sorbents (MEPS) carried out by the EPREP company (Victoria, Australia). The μSPEed is a solid-phase-based extraction procedure miniaturized in which the extraction cartridge contains a one-way pressure-driven valve to withdraw the sample flow in a single direction. This is the main difference with the MEPS technique, in which there is a two-directional flow potential (up and down) through the sorbent.^29,30^ In this sense, in μSPEed, thanks to the valve, the aspiration of the sample or the solvents is achieved by means of vacuum when the plunger is pulled back, so the flow does not pass through the sorbent bed as in MEPS; instead it bypasses the sorbent. In addition, this configuration enables constant and high pressure (up to 1600 psi) flows, providing efficient extraction of the analytes. Another advantage is that μSPEed uses smaller sorbent particles of <3 μm, instead of the 50–60 μm particles normally used in MEPS. These smaller particles provide higher surface area, and consequently, a more efficient extraction of the analytes.^30^

Although this technique is not new, to date, it has been scarcely applied in food analysis despite providing quick procedures with high extraction potential, great efficiency, and simplicity. So far, μSPEed has been successfully applied for the extraction of phenolic acids from tea,^31^ extraction of polyphenols from baby foods,^32^ trihalomethane disinfection byproducts in water,^33^ and for on-column derivatization of short-chain fatty acids in olive oil prior to extraction.^34^

Hence, the aim of this work was to evaluate the suitability of the innovative μSPEed technique to perform a multicomponent extraction of PAs followed by their analysis by ultrahigh-performance liquid chromatography coupled to ion-trap tandem mass spectrometry (UHPLC-IT-MS/MS) in order to propose a sustainable and sensitive analytical methodology to monitor the occurrence of these alkaloids in prepared individual infusions of mallow, calendula, and hibiscus flowers, in which the evaluation of PAs has been scarcely studied. Moreover, the determination of PAs was directly performed in the infusion samples instead of the dry flowers, in order to perform an estimation of the real exposure of consumers to these contaminants when they drink this type of products with therapeutic or dietetic purposes. To the best of our knowledge, this is the first time that μSPEed is used for the determination of PAs and PANOs in food samples or other matrices.

## 2 Materials and Methods

### 2.1 Chemicals, Materials, and Standard Solutions

Methanol (MeOH), acetonitrile LC–MS grade, dimethyl sulfoxide (DMSO), and ammonia solution 32% were purchased from Scharlab (Barcelona, Spain). Ammonium acetate and formic acid LC–MS grade were acquired from Fluka (Busch, Switzerland). Milli-Q water (resistivity 18.2 MΩ cm) was obtained from a Millipore Milli-Q System (Billerica, MA, USA). The μSPEed procedure was carried out with an electronic digiVol digital syringe (250 μL) acquired from EPREP (Mulgrave, Victoria, Australia). μSPEed cartridges: silica (3 μm, 120 Å), octadecylsilane (C18 silica-based, 3 μm, 120 Å), porous crosslinked polystyrene divinyl benzene (PS/DVB, 3 μm, 300 Å), and porous phenyl crosslinked polystyrene divinyl benzene (PS/DVB-RP, 3 μm, 300 Å) were also obtained from EPREP (Mulgrave, Victoria, Australia).

Standards of PAs and related PANOs with high purity grade (≥90%) were acquired from PhytoLab GmbH & Co. KG (Vestenbergsgreuth, Germany). Only retrorsine was from Sigma-Aldrich (St. Louis, MO, USA). Individual standard solutions (1000 μg/mL) of each compound were prepared according to their solubility. Accordingly, europine, europine *N*-oxide, heliotrine, heliotrine *N*-oxide, intermedine, lycopsamine, retrorsine, senecionine, and seneciphylline were prepared in acetonitrile/DMSO (4/1, v/v), whereas echimidine, echimidine *N*-oxide, intermedine *N*-oxide, lasiocarpine, lasiocarpine *N*-oxide, lycopsamine *N*-oxide, retrorsine *N*-oxide, seneciphylline *N*-oxide, senecionine *N*-oxide, senecivernine, senecivernine *N*-oxide, and senkirkin were prepared in MeOH. From the individual solutions, a mix-standard solution containing all the 21 analytes at 1 μg/mL (each of them) was prepared in water. This multicomponent solution was used to achieve working standard solutions in MeOH at different concentration levels to develop, optimize, and validate the analytical performance of the method. All the standard solutions were stored at −20 °C.

### 2.2 Samples and Preparation of Infusions

Edible dried flowers, including mallow (*Alcea rosea*, plant family Malvaceae), calendula (*Calendula officinalis*, plant family Asteraceae), and hibiscus (*Hibiscus sabdariffa*, plant family Malvaceae), were acquired in bulk bags at different stores from Spain and Portugal. Sampling was performed according to the European Commission Regulation No. 401/2006 concerning sampling and analysis of mycotoxins in foodstuff.^35^ Hence, three subsamples were acquired for each lot number. Sample details are shown in Table S1. Samples were denoted by indicating in the first letter the type of flower (*M* for mallow, *C* for calendula, and *H* for hibiscus).

Infusions of the flowers were performed according to the manufacturers’ instructions to resemble the real conditions that consumers carry out in the culinary preparation of these products when they acquire them. In this sense, 5 g of dried flowers was weighed in an analytical balance (±0.1 mg) and infused with 200 mL of boiling water (100 °C), allowing brewing for 10 min (Figure S1). Then, the infusion was strained and kept at 4 °C until analysis. Before extraction, the samples were filtered through a 0.45 μm PTFE filter membrane. Each subsample was infused in triplicate, and each infusion extract was analyzed in triplicate.

### 2.3 μSPEed Extraction Procedure

**Figure 1:**
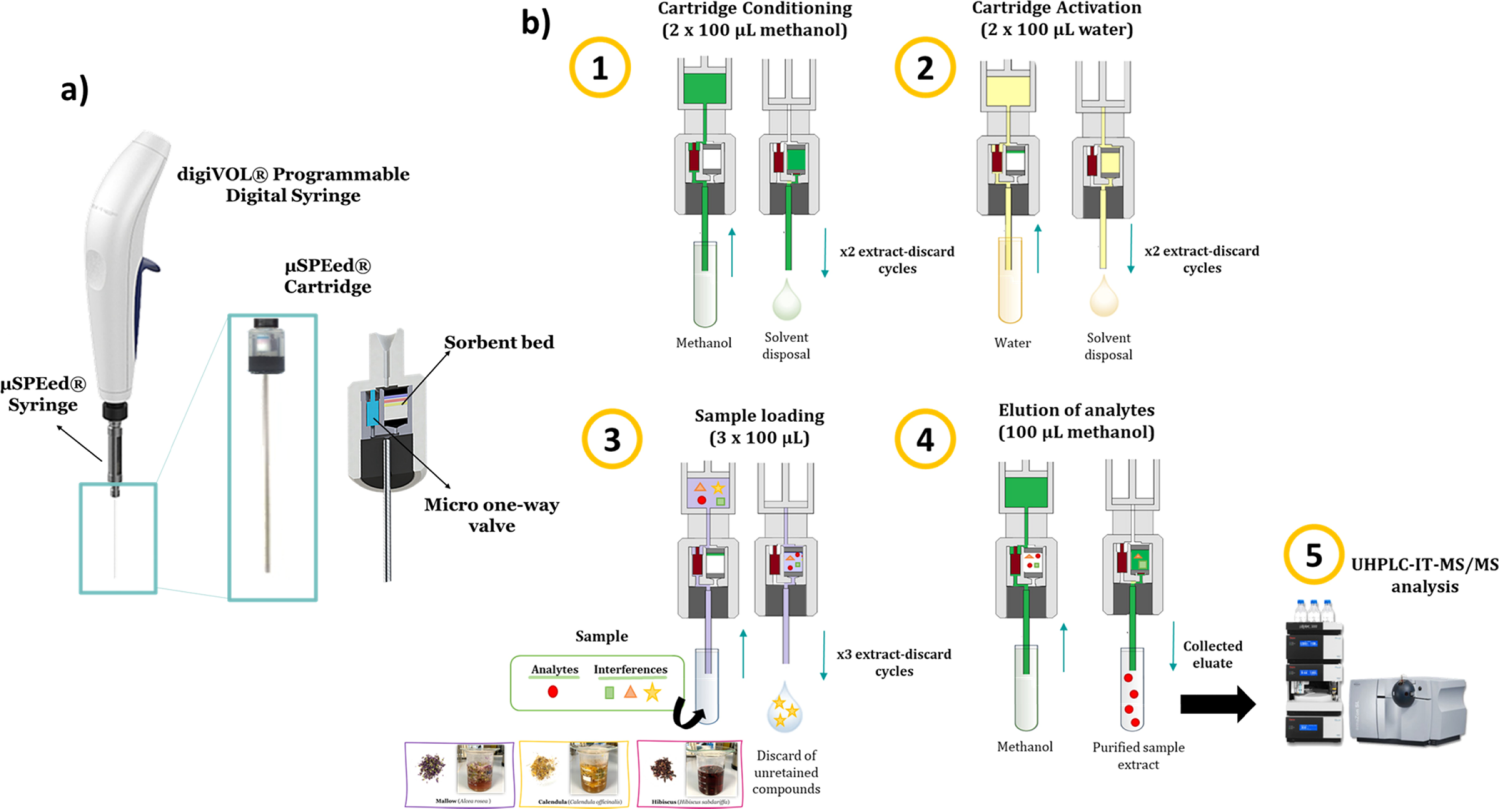
Graphical scheme of (a) μSPEed configuration and (b) experimental procedure proposed for extraction under the optimized conditions.

Under the optimized conditions, the extraction of the flower infusion extracts was carried out with the μSPEed digital syringe (in the extract-discard mode) using the C18 sorbent as follows: the sorbent was first conditioned with two aspiration–dispense cycles of 100 μL of MeOH followed by two aspiration–dispense cycles of 100 μL of water. Then, for sample loading, three aspiration–dispense cycles of 100 μL of the infusion extract were passed through the syringe. In μSPEed, sample loading can be performed in two different modes: draw-eject (the sample volume aspirated is discarded in the same vial of the sample after each extraction cycle) or extract-discard (the sample volume aspirated is discarded in a waste vial after each extraction cycle). Accordingly, the extract-discard mode was chosen for sample loading. No washing step was performed, so after the loading step, the analytes were directly eluted from the sorbent with 100 μL of MeOH into a vial for its subsequent chromatographic analysis. Between each extraction, the cartridge was rinsed with 4 × 100 μL of MeOH to avoid memory effects (carry-over) and to act as a conditioning step before the next extraction. The aspiration–dispense flow rate was automatically set in all assays to 20 μL/s to avoid cavitation. Figure 1 schematically shows the experimental layout described and performed under the optimized conditions.

### 2.4 UHPLC-IT-MS/MS Analysis

The chromatographic analysis of the sample extracts was performed with an UHPLC system (Dionex UltiMate 3000, Thermo Scientific, Waltham, MA, USA) coupled to an ion-trap tandem mass spectrometer detector (ESI-ITMS amaZon SL, Bruker, Billerica, MA, USA) and using a Luna Omega Polar C18 column (100 mm × 2.1 mm, 1.6 μm particle size, Phenomenex, Torrance, CA, USA) at 25 °C. The separation of the analytes was achieved using a mobile phase gradient elution, which was carried out by combining MeOH containing 10 mM ammonium acetate (solvent A) and water containing 5 mM ammonium acetate and 0.2% formic (solvent B): 5% A (0–0.5 min), 5–50% A (0.5–7 min), 50% A (7–7.5 min), 50–100% A (7.5–11 min), 100% A (11–12 min), 100–5% A (12–14 min), and 1 min for re-equilibration to initial conditions, yielding a total analysis time of 15 min. The injection volume was 5 μL, and the flow rate was set constant to 0.250 mL/min. Under these conditions, the chromatographic separation of the 21 PAs/PANOs established as mandatory in the legislation was achieved within 10 min (Figure S2).

For mass spectrometry acquisition, the electrospray ionization interface (ESI) was used in positive ion mode. The end plate offset was set at −500 V, the capillary voltage at −4500 V, the nebulizer gas at 20 psi, the dry gas at 10 L/min, and the dry temperature at 200 °C. Multiple reaction monitoring scan mode was used for all analytes. To achieve the mass spectrum parameters of each analyte, individual standard solutions (5 μg/mL) of PAs were directly infused at a flow rate of 4 μL/min in the ESI source. This way it was possible to identify the precursor ion of each analyte ([M + H]^+^), which was then isolated and fragmentated to obtain the mass spectrum (MS^2^) with the corresponding product ions of each analyte. The most intense product ion obtained in the MS^2^ spectrum of each analyte was selected for quantification, whereas the others were used for qualitative identification purposes (Figure S2).

### 2.5 Analytical Validation of the Method

The extraction procedure proposed using the μSPEed technique was validated in terms of accuracy, precision, linearity, matrix effects (ME), and limits of detection and quantification for each type of flower matrix. In this sense, samples M-1, C-1, and H-1 (Table S1) were used for the validation determinations of mallow, calendula, and hibiscus matrices, respectively. Currently, there is no official regulation for the validation of analytical procedures regarding the determination of PAs and PANOs in food or feed, so these analytical parameters were evaluated according to the criteria set in the European Commission SANTE/12682/2019 document and in regulation EC No 401/2006.^35,36^ Nonetheless, there is a regulation that establishes maximum levels of PAs in certain foodstuffs.^27^ According to this document, the maximum concentration allowed for dried products intended for herbal infusions such as edible dried flowers (except rooibos, anise, lemon balm, chamomile, thyme, peppermint, lemon verbena, and mixtures exclusively composed of these dried herbs) is 200 μg/kg. Therefore, considering the amount of dried flower used for the infusions (5 g) and the water volume (200 mL), 200 μg/kg corresponds to a concentration of 5 μg/L in the infusion considering a 100% transfer rate for all the target PAs. Since previous studies have evaluated transfer rates of PAs to infusions of about 80–100%,^24−26^ this concentration value was set as intermediate level for validation purposes.

Based on these concentration values, and according to the sensitivity achieved for each analyte in the UHPLC-IT-MS/MS (Figures S3–S5), a compromise was reached to choose 1 μg/L (corresponding to 40 μg/kg) and 50 μg/L (corresponding to 2000 μg/kg) as low and high validation levels, respectively. In this sense, the accuracy was assessed for each matrix at the three concentration levels indicated above, and it was determined in terms of recovery. For this purpose, recovery assays were carried out by spiking the infusion samples at the different concentration validation levels and, afterward, subjecting them to the microextraction procedure. The areas obtained from the chromatographic analysis of these sample extracts were then compared with the areas obtained from the analysis of simulated sample extracts (nonspiked infusion samples subjected to the microextraction procedure and spiked afterward their extraction at the same concentration level before their chromatographic analysis). The results were expressed as the mean recovery obtained from nine samples (*n* = 9) extracted in different days. According to the validation guidelines, the recovery values should be between 70 and 120%.^35,36^

Likewise, method precision (expressed as relative standard deviation percentage, RSD%) was assessed for each matrix at the same validation levels used for the accuracy (low, intermediate, and high), and it was evaluated in terms of intraday (repeatability) and interday (reproducibility) precision. Intraday precision was achieved from the analysis of six replicate extracts (*n* = 6) obtained on the same day from an infusion sample spiked with the analytes at the corresponding validation level tested. Interday precision was determined through the analysis of three replicate extracts of a sample (spiked with the analytes at the corresponding validation level), which were carried out throughout three different days (*n* = 9). According to the validation recommendations, RSD values for the precision parameters should be ≤20%.^35,36^

Linearity for each flower matrix was determined with matrix-matched calibration curves, which were prepared for each matrix at six known concentration levels within the linear range evaluated (1–100 μg/L). For this purpose, the sample extracts obtained after the μSPEed procedure were spiked with an aliquot of a standard solution containing all the target analytes according to the desired concentration of the calibration curve. Additionally, in case analytes could occurred in the flower matrix in a natural way, an unspiked sample extract (called blank sample) was also subjected to the microextraction procedure and analyzed, so the analyte signal could be subtracted for correction purposes. According to the validation guidelines, good linearity involves achieving coefficient of determination (*R*^2^) values closed to 1.^35,36^ On the other hand, solvent-based calibration curves prepared with working standard solutions at the same concentration levels as the matrix-matched calibration curves and not subjected to the μSPEed procedure were carried out to determine ME. For this purpose, the slopes of the calibration equations obtained for each analyte from both matrix-matched and solvent-based calibration curves (both expressed in the same units μg/L) were compared, and ME was calculated as follows: [(slope matrix-matched/slope solvent-based) – 1] × 100. Positive values indicate signal increase, while negative values mean signal suppression. ME within ±20% can be ignored, and matrix-matched calibration curves can be avoided for analyte quantification. Conversely, values without this range must be considered in calibration.^36^ Likewise, when ME values are between −50% < MEs < −20% and 50% > MEs > 20%, it can be considered a soft effect, while values below −50% or above 50% are considered as a strong effect.^37,38^

For method selectivity, the spectra of the different analytes obtained from standard solutions were compared with the spectra obtained in the samples. Following the validation criteria, it is considered satisfactory when variations in the spectra are less than ±30% and the retention time of the analytes is within the interval ±2.5%.^36^ On the other hand, the sensitivity of the method was established through the method detection limits (MDLs) and method quantification limits (MQLs) of the analytes in each matrix. These limits were estimated based on the signal-to-noise ratio (S/N) provided by the UHPLC-IT-MS/MS software from the extracted ion chromatograms of each analyte in each flower matrix (Figures S3–S5). Accordingly, the concentrations yielding a S/N of 3 and 10 were considered for the MDLs and MQLs, respectively. The S/N was corrected by subtracting the signal obtained from the blank samples (not spiked) on those analytes which were detected in the samples analyzed.

## 3 Results and Discussion

### 3.1 Evaluation of Extraction Conditions

To establish the most suitable and sustainable extraction conditions for the μSPEed procedure, several parameters were first evaluated, such as the type of sorbent, the sample pH, the washing step, the number of extraction cycles, and the elution volume. All assays were performed in triplicate for each optimized extraction parameter, and the extraction efficiency was determined by the total peak area response observed in the chromatographic system, or by recovery assays.

First, the type of sorbent was evaluated. Strong-cation-exchange (SCX) sorbents have been extensively used for the purification of PAs from food samples, followed by reversed-phase sorbents (mainly based on C18) and mixed-mode sorbents (combination of reversed-phase and cation-exchange interactions) in solid-phase extraction (SPE).^2^ Although mixed-mode sorbents improve selectivity and provide different types of interactions, they are not currently available for μSPEed cartridges. On the other hand, μSPEed cartridges with SCX sorbents are commercially available, but they were not evaluated because they require greater pH control throughout the analytical performance, as pH must be correctly adjusted in every step of the extraction procedure to achieve a correct interaction between the analytes and the sorbent and afterward their complete desorption from the sorbent with a different pH. These pH changes imply more time, as well as the introduction of acids or bases that lead to dirtier extracts that are then injected in the chromatographic system and in the mass spectrometer (such as ammonium or sodium salts). The injection of these dirtier extracts can produce loss of sensitivity and precipitation problems in the chromatographic column. For these reasons, C18 sorbents were preferred. Therefore, the sorbents evaluated were silica, C18, PS/DVB, and PS/DVB-RP. They were selected based on their availability for μSPEed cartridges and to explore other alternative sorbents and see their possible extraction potential for this type of analytes.

The sorbents selected were first evaluated using standard solutions in water (50 μg/L of each analyte) at different pH conditions (nonbasified medium and medium adjusted to pH 10.0 with ammonia solution). These sorbents were tested under preliminary extraction conditions as follows: conditioning step of the sorbent with two aspiration–dispense cycles of 100 μL of MeOH followed by two aspiration–dispense cycles of 100 μL of water. Then, sample loading with five aspiration–dispense cycles of 100 μL of the standard solutions. Afterward, a washing step with one aspiration–dispense cycle of 100 μL of water and finally elution into the chromatographic vial with two aspiration–dispense cycles of 100 μL of MeOH (final elution volume = 200 μL). Therefore, all the eluted extracts were injected in the same medium (nonbasified methanol), so they could be compared.

**Figure 2:**
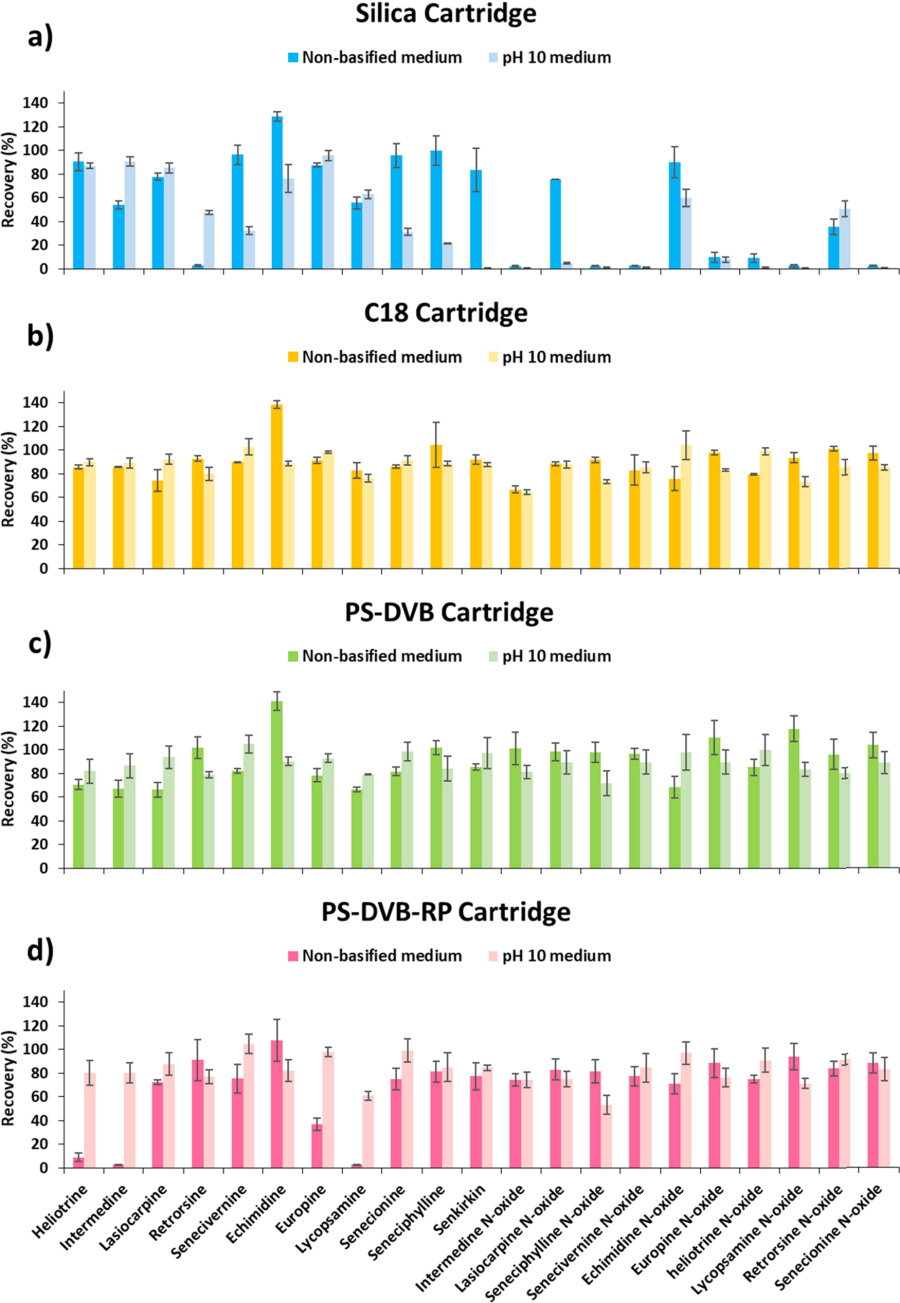
Recovery values obtained with (a) silica, (b) C18, (c) PS/DVB, (d) PS/DVB-RP cartridges from the μSPEed analysis of standard solutions in water (50 μg/L of each analyte) at different pH extraction conditions (nonbasified and basified media). Extraction conditions: cartridge conditioning with 2 × 100 μL methanol and 2 × 100 μL cycles; 5 × 100 μL sample loading; washing with 100 μL water; elution with 2 × 100 μL methanol.

As can be observed in Figure 2, the silica cartridge provided the worst extraction efficiency toward the target analytes at both extraction conditions, as many compounds showed very low recovery values (1–5%). On the other hand, among the other cartridges, C18, PS/DVB, and PS/DVB-RP showed a similar extraction efficiency at pH 10.0 providing recoveries in an acceptable range (64–105%). However, PS/DVB-RP cartridge showed to be less effective at these conditions for some compounds, such as seneciphylline *N*-oxide (53%) and lycopsamine (61%). Moreover, very low recoveries (3–37%) were achieved for several analytes with the PS/DVB-RP cartridge under the nonbasified extraction conditions (heliotrine, intermedine, lycopsamine, and europine). Conversely, regarding the recovery values obtained with C18 and PS/DVB cartridges, in general, no big differences were observed among the different pH extraction conditions or even among the two cartridges. Therefore, among the four sorbents tested, the C18 and PS/DVB cartridges were selected to further evaluate their extraction efficiency in the flower sample matrices.

**Figure 3:**
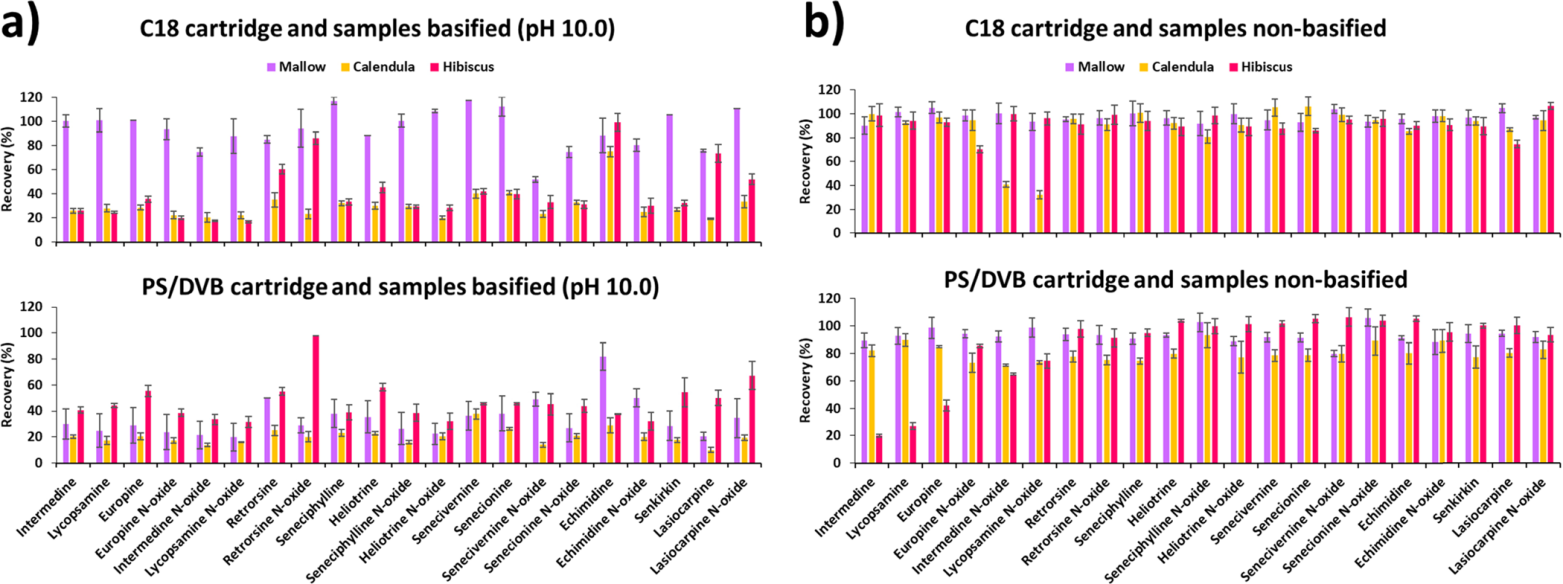
Recovery values obtained with C18 and PS/DVB cartridges from the μSPEed analysis of mallow, calendula and hibiscus infusions spiked with the analytes (50 μg/L of each analyte) and (a) basified at pH 10.0 and (b) nonbasified before extraction. Extraction conditions: cartridge conditioning with 2 × 100 μL methanol and 2 × 100 μL cycles; 5 × 100 μL sample loading; washing with 100 μL water; elution with 2 × 100 μL methanol.

Accordingly, the same extraction procedure described above was used to extract the PAs from the flower infusions of mallow, calendula, and hibiscus spiked with the analytes at 50 μg/L (of each analyte) and extracted at different pH conditions (nonbasified medium and medium adjusted to pH 10.0 with ammonia solution) using both C18 and PS/DVB cartridges. As Figure 3a shows, low recoveries were achieved with both cartridges when the infusions were basified to pH 10.0 before extraction, except for some analytes in the case of mallow with the C18 sorbent. Conversely, better results were achieved when the infusions were not basified before extraction (Figure 3b), so it was decided not to basify the infusions in following trials. This may be due to the occurrence of other components of the matrices, such as polyphenols, which may have stronger affinity for the active sites of the sorbents than the PAs at basic pH, leading to lower recovery values than in the standard solutions. Likewise, it was observed that, in general, better recovery values were achieved with the C18 sorbent than with the PS/DVB cartridge, mainly in the case of the hibiscus matrix (Figure 3b). Therefore, although the recovery values obtained for some analytes (intermedine *N*-oxide and lycopsamine *N*-oxide) were lower than 60% in the calendula matrix with C18 (Figure 3b), to reach a compromise among the three matrices, the C18 sorbent was selected as the best option to perform the microextraction of the 21 PAs/PANOs. Moreover, this sorbent is cheaper than the PS/DVB cartridge.

After selecting the cartridge sorbent, the number of extraction cycles (3 and 5 extract-discard cycles) was evaluated, considering in both cases the washing step as well as its omission. In general, the results obtained showed that in the case of mallow, the washing step did not have a big effect on the extraction efficiency of the analytes at both 3 and 5 extraction cycles (Figure S6). Conversely, in calendula and hibiscus matrices, the recovery values were in general higher with 5 extraction cycles, including the washing step than without it (Figures S7 and S8). However, in calendula, the recovery of intermedine *N*-oxide and lycopsamine *N*-oxide slightly improved without the washing step. This suggest that the extraction efficiency of these analytes can be influenced by this step, promoting their early coelution due to their polar characteristics. In fact, the same effect was observed in more analytes using 3 extraction cycles in the calendula matrix (Figure S7). Likewise, the results obtained showed that with 3 extraction cycles, the recoveries of the analytes were in general better without performing the washing step in the hibiscus matrix (Figure S8). On the other hand, although with 5 extraction cycles the recovery values of some analytes were better, the recoveries obtained in the 3 matrices with 3 extraction cycles were in all cases higher than 70% (Figure S9), which are acceptable values for method performance.^35^ Therefore, to save time and reduce the volume of samples and organic solvents used in the method, it was decided to skip the washing step and perform 3 extraction cycles, as good and valid recoveries were achieved under these extraction conditions.

Finally, different elution volumes of MeOH (100, 200, and 250 μL) were evaluated in the three flower matrices using 3 extraction cycles and without the washing step. No big differences were observed among the different elution volumes tested, but in general, higher recovery values of the analytes were achieved with 250 μL in all three matrices (Figure S10). Nevertheless, it was observed that 100 μL of MeOH was enough to elute all the analytes and achieve acceptable recovery values for method performance (≥70%),^35^ obtaining values in the range 76–100, 73–95, and 71–107% for mallow, calendula, and hibiscus, respectively (Figure S10).

Hence, as good recoveries were obtained and in order to reduce the volume of the organic solvents employed in the method, it was decided to choose 100 μL of MeOH for the elution step. Therefore, the overall final experimental conditions for the μSPEed procedure were C18 sorbent, 3 draw-eject extraction cycles (3 × 100 μL of sample nonbasified), and elution with 100 μL of MeOH without prior washing step (Figure 1). Under these conditions selected, the final extraction procedure took less than 1 min per sample, and only 300 μL of organic solvent MeOH and 300 μL of the sample were required per extraction, leading to an environmentally friendly analytical method for the extraction of PAs.

Table 1 shows a comparison of the proposed μSPEed method with other published methods that performed determination of PAs in teas and herbal tea samples. As it can be observed, the great majority of them used SPE technique for the extraction and purification of PAs in which significantly higher volumes of sample and organic solvents are required. Likewise, is extremely time-consuming in comparison to μSPEed and the amounts of sorbents used are greater, from 30 to 500 mg compared to the 4 mg of the μSPEed cartridge. For these reasons, the μSPEed technique is an improved miniaturized format of conventional SPE that provides multiple advantages over it, mainly quicker extractions and less solvent consumption. The smaller particle size sorbents used in the μSPEed enable faster extraction with elution in narrow precise band, and the possibility of using less solvent allows achieving a very high concentration factor, which avoids performing a subsequent evaporation step, as usually reported in previous studies (Table 1).

**Table 1:** Comparison of the Proposed μSPEed Method with Other Published Methods that Performed Determination of Pyrrolizidine Alkaloids in Teas and Herbal Infusion Samples^a^

number of PAs	sample/extract^b^	extraction/purification procedure (sorbent amount) and time estimated^c^	organic solvents employed (mL)	analysis	MQL	recovery (%)	precision (RSD, %)	range of PAs content found	ref.
21	2 mL sample extract	SPE (150 mg) and 48 min + evaporation	48 mL	UHPLC–MS/MS	0.3–9.0 μg/kg	87–101	0.08–4.82	0–1.88 mg/kg	(11)
31	40 mL infusion	SPE (500 mg) and 121 min + extract evaporation	19 mL	UHPLC-TQ-MS/MS	0.01 μg/L	96–113	1.71–35	0.1–187,151 μg/kg	(12)
20	50 mL infusion	SPE (500 mg) and 171 min + extract evaporation	16.27 mL	UHPLC-TQ-MS/MS	0.07–0.14 μg/L	88–116	0.6–9.6	0–311 μg/kg	(13)
14	2.5 mL sample extract	SPE (60 mg sorbent) and > 15 min + extract evaporation	23 mL	HPLC-TQ-MS/MS	1.3–6.3 μg/kg	93–127	3–19	10–1733 μg/kg	(14)
17	10 mL sample extract	SPE (500 mg sorbent) and > 30 min + extract evaporation	56 mL	HPLC-TQ-MS/MS	2–6.4 μg/kg	45–122	1–20	0–5647 μg/kg	(15)
16	5 g dry sample	SLE and 60 min	30 mL	UHPLC-TQ-MS/MS	0.07–0.73 μg/kg	80–97		0.08–314 μg/kg	(16)
11	1 g dry sample	QuEChERS (5 g partitioning salts +0.4 g dispersive clean-up sorbents) and 71 min	20 mL	HPLC-Q-Orbitrap-MS/MS	≥1–100 μg/kg	70–112	0.25–14		(17)
28	1 g dry sample	SLE and 40 min + extract evaporation	10 mL	HPLC-QTRAP-MS/MS	10–50 μg/kg	80–95	0.6–8.5	20–1729 μg/kg	(18)
23	10 mL sample extract	SPE (500 mg sorbent) and 115 min + extract evaporation	36 mL	HPLC-TQ-MS/MS	10 μg/kg	76–125		0–5668 μg/kg	(19)
								13–1080 μg/kg^d^	
28	20 mL sample extract	SPE (500 mg) and 17 h + extract evaporation	78 mL	UHPLC-QTRAP-MS/MS	0.015–0.075 μg/kg	85–116	3.2–13-4		(20)
25	10 mL sample extract	SPE (500 mg) and 82 min + extract evaporation	52 mL	HPLC-TQ-MS/MS	0.61–5.40 μg/kg	49–114	0.6–37.4		(21)
44	10 mL sample extract	SPE (500 mg) and 59 min + extract evaporation	62 mL	HPLC-TQ-MS/MS	0.1–27.9 μg/kg	52–152	0.7–16.1	0.1–47.9 μg/kg	(22)
28	1 g dry sample	QuEChERS (7.5 partitioning salts +2400 mg dispersive clean-up sorbents) and 32 min + extract evaporation	30 mL	HPLC-Q-Orbitrap-MS/MS	5 μg/kg	87–111	6–20		(23)
38	50 mL infusion	SPE (500 mg) and 160 min + extract evaporation	16 mL	UHPLC-TQ-MS/MS		45–122	1–20	1394–4805 μg/L	(25)
37	17–300 mL infusion	SPE (30–200 mg sorbent) and > 300 min + extract evaporation	>18.5 mL	HPLC-QToF-MS/MS		30–98	3–17	154–2412 μg/kg	(26)
70	2 g dry sample			UHPLC-TQ-MS/MS	0.05 μg/L	73–107	3.1–24	30.7–1120 μg/L	(39)
21	300 μL infusion	μSPEed® (4 mg sorbent) and 1 min	300 μL	UHPLC-IT-MS/MS	0.3–1.0 μg/L	79–97	1–17	23–113 μg/L	This work
								920–4520 μg/kg^d^	

a HPLC: High performance liquid chromatography; IT: ion-trap; LOQ: limit of quantification; MS: mass spectrometry; MS/MS: tandem mass spectrometry; PAs: pyrrolizidine alkaloikds; Q: single quadrupole; QToF: quadrupole time-of-flight; QTRAP: hybrid triple quadrupole-linear ion trap; QuEChERS: quick, easy, cheap, effective, rugged, and safe; SLE: solid–liquid extraction; SPE: solid-phase extraction; TQ: triple quadrupole; UHPLC: ultrahigh performance liquid chromatography; MQL: method quantification limit.

b Extract refers to the volume of sample extract used in the purification step.

c For time estimation, a flow rate of 1 mL/min was considered in SPE procedures, when not indicated the flow rate.

d Estimation of the pyrrolizidine alkaloids content in the dry product considering the concentration found in the infusion samples and transfer efficiency of 100%.

Other works have also used the QuEChERS (acronym of quick, easy, cheap, effective, rugged, and safe) strategy (Table 1). However, also higher amounts of sorbents and partitioning salts, as well as organic solvents are required than in μSPEed (Table 1), besides being more tedious (several agitation and centrifugation steps) and time-consuming. On the other hand, Chen et al. did not perform previous extraction of PAs from the dry tea samples; they directly prepared the infusion and injected an aliquot of the sample into the chromatographic system.^39^ However, when using a mass spectrometer detector, it is not convenient to directly inject the sample extracts without a clean-up or purification procedure, especially if there are matrix interferences, as it can foul the ionization source and decrease the sensitivity of the equipment, leading to more frequent and thorough expensive maintenance of the detector. Therefore, overall, the μSPEed is a very suitable and potential technique for the direct extraction and purification of liquid samples, such as teas and herbal infusions, which leads to the development of quick, sensitive, selective, environment-friendly, and cost-effective methods for the analysis of these beverages. Finally, the method proposed here can be easily scaled to automatic and high-throughput systems using the ePrep Sample Preparation Workstation (EPREP, Australia).

### 3.2 Method Validation

The validation parameters evaluated in the three matrices are presented in Tables 2^3^,4, showing the good analytical performance of the method developed. As it can be observed, good linear regression was achieved for all analytes in the three matrices over the range of concentrations studied, providing by least-squares linear regression analysis excellent coefficient of determination (*R*^2^) values higher than 0.990, which ranged between 0.995–0.999, 0.995–0.999, and 0.993–0.999 for mallow, calendula, and hibiscus, respectively (Tables 2^3^,4).

**Table 2:** Validation Parameters of the μSPEed Procedure Proposed for the Determination of the Target PAs/PANOs in Mallow Infusion Samples^a^

analytes	linearity (*R*^2^)	accuracy		precision		MDL (μg/L)	MQL (μg/L)	ME (%)
		recovery	mean recovery	intraday	interday			
		(% ± SD)	(% ± SD)	(RSD%)	(RSD%)			
intermedine	0.998	89 ± 6^b^	86 ± 9	4^b^	7^b^	0.3	1.0	6
		94 ± 9^c^		9^c^	9^c^			
		76 ± 6^d^		7^d^	12^d^			
lycopsamine	0.999	88 ± 4^b^	88 ± 7	5^b^	5^b^	0.3	1.0	9
		94 ± 6^c^		4^c^	6^c^			
		81 ± 5^d^		7^d^	8^d^			
europine	0.998	90 ± 7^b^	86 ± 6	3^b^	8^b^	0.3	1.0	42
		80 ± 10^c^		8^c^	13^c^			
		89 ± 7^d^		8^d^	12^d^			
europine *N*-oxide	0.998	97 ± 6^b^	90 ± 9	4^b^	7^b^	0.2	0.6	10
		92 ± 8^c^		3^c^	9^c^			
		80 ± 9^d^		11^d^	12^d^			
intermedine *N*-oxide	0.997	89 ± 2^b^	82 ± 7	2^b^	11^b^	0.3	1.0	–29
		82 ± 10^c^		9^c^	12^c^			
		75 ± 5^d^		6^d^	13^d^			
lycopsamine *N*-oxide	0.997	77 ± 10^b^	81 ± 6	6^b^	13^b^	0.3	1.0	44
		88 ± 9^c^		7^c^	11^c^			
		79 ± 5^d^		6^d^	14^d^			
retrorsine	0.999	97 ± 5^b^	86 ± 10	5^b^	6^b^	0.3	1.0	–53
		82 ± 8^c^		10^c^	10^c^			
		78 ± 8^d^		5^d^	11^d^			
retrorsine *N*-oxide	0.999	95 ± 10^b^	89 ± 9	6^b^	11^b^	0.3	1.0	–22
		92 ± 12^c^		3^c^	12^c^			
		79 ± 4^d^		5^d^	5^d^			
seneciphylline	0.998	84 ± 7^b^	83 ± 3	7^b^	8^b^	0.3	1.0	–77
		85 ± 7^c^		8^c^	9^c^			
		79 ± 6^d^		7^d^	7^d^			
heliotrine	0.996	86 ± 8^b^	85 ± 2	9^b^	9^b^	0.3	0.9	–47
		83 ± 6^c^		7^c^	8^c^			
		85 ± 10^d^		12^d^	12^d^			
seneciphylline *N*-oxide	0.998	85 ± 8^b^	81 ± 5	6^b^	9^b^	0.3	1.0	–18
		76 ± 10^c^		10^c^	13^c^			
		83 ± 8^d^		9^d^	9^d^			
heliotrine *N*-oxide	0.999	85 ± 7^b^	86 ± 2	5^b^	9^b^	0.3	1.0	–62
		85 ± 8^c^		5^c^	9^c^			
		88 ± 9^d^		10^d^	14^d^			
senecivernine	0.999	102 ± 7^b^	91 ± 10	7^b^	9^b^	0.2	0.7	–59
		89 ± 9^c^		8^c^	10^c^			
		82 ± 8^d^		9^d^	10^d^			
senecionine	0.999	80 ± 9^b^	81 ± 3	7^b^	12^b^	0.2	0.7	–65
		85 ± 11^c^		9^c^	13^c^			
		79 ± 4^d^		6^d^	13^d^			
senecivernine *N*-oxide	0.995	92 ± 8^b^	91 ± 2	6^b^	9^b^	0.3	1.0	–24
		89 ± 5^c^		3^c^	6^c^			
		91 ± 6^d^		7^d^	8^d^			
senecionine *N*-oxide	0.998	74 ± 8^b^	79 ± 5	11^b^	13^b^	0.3	1.0	–37
		79 ± 9^c^		8^c^	12^c^			
		83 ± 9^d^		10^d^	11^d^			
echimidine	0.995	86 ± 8^b^	90 ± 9	9^b^	9^b^	0.2	0.5	–59
		83 ± 9^c^		8^c^	10^c^			
		100 ± 8^d^		6^d^	8^d^			
echimidine *N*-oxide	0.997	88 ± 7^b^	90 ± 4	8^b^	8^b^	0.3	1.0	2
		94 ± 9^c^		4^c^	9^c^			
		87 ± 9^d^		6^d^	10^d^			
senkirkin	0.996	90 ± 5^b^	84 ± 6	4^b^	5^b^	0.3	1.0	–10
		78 ± 5^c^		6^c^	6^c^			
		83 ± 9^d^		11^d^	11^d^			
lasiocarpine	0.995	92 ± 11^b^	90 ± 6	9^b^	12^b^	0.3	1.0	–60
		94 ± 5^c^		5^c^	7^c^			
		83 ± 9^d^		9^d^	10^d^			
lasiocarpine *N*-oxide	0.994	86 ± 7^b^	87 ± 7	7^b^	8^b^	0.2	0.7	–71
		80 ± 12^c^		7^c^	14^c^			
		94 ± 9^d^		10^d^	11^d^			

a Recovery: mean recovery obtained from nine samples (*n* = 9) spiked with the analytes at a known concentration level, and subjected to the proposed extraction procedure; intraday precision: six replicate extracts (*n* = 6) analyzed on the same day of an infusion sample spiked with the analytes at a known concentration level; interday precision: three replicates extracts of a sample analyzed throughout three different days (*n* = 9) and spiked with the analytes at a known concentration level; MDL: method detection limit; MQL: method quantification limit; ME: matrix effect.

b Low spiked level (1 μg/L of flower infusion).

c Medium spiked level (5 μg/L).

d High spiked level (50 μg/L).

**Table 3:** Validation Parameters of the μSPEed Procedure Proposed for the Determination of the Target PAs/PANOs in Calendula Infusion Samples^a^

analytes	linearity (*R*^2^)	accuracy		precision		MDL (μg/L)	MQL (μg/L)	ME (%)
		recovery	mean recovery	intraday	interday			
		(% ± SD)	(% ± SD)	(RSD%)	(RSD%)			
intermedine	0.995	82 ± 1^b^	85 ± 12	1^b^	13^b^	0.3	1.0	–24
		99 ± 6^c^		3^c^	6^c^			
		75 ± 4^d^		5^d^	15^d^			
lycopsamine	0.999	85 ± 8^b^	92 ± 6	7^b^	10^b^	0.3	1.0	6
		96 ± 5^c^		4^c^	5^c^			
		94 ± 9^d^		6^d^	9^d^			
europine	0.995	73 ± 9^b^	85 ± 12	10^b^	11^b^	0.2	0.5	62
		97 ± 8^c^		6^c^	8^c^			
		85 ± 12^d^		6^d^	14^d^			
europine *N*-oxide	0.999	103 ± 6^b^	97 ± 15	3^b^	6^b^	0.2	0.7	40
		107 ± 9^c^		8^c^	10^c^			
		80 ± 8^d^		10^d^	14^d^			
intermedine *N*-oxide	0.999	94 ± 9^b^	87 ± 12	3^b^	10^b^	0.3	1.0	–21
		94 ± 5^c^		6^c^	7^c^			
		73 ± 5^d^		7^d^	10^d^			
lycopsamine *N*-oxide	0.995	78 ± 3^b^	84 ± 6	4^b^	13^b^	0.3	1.0	–7
		89 ± 6^c^		7^c^	7^c^			
		84 ± 5^d^		6^d^	7^d^			
retrorsine	0.996	74 ± 7^b^	83 ± 10	9^b^	11^b^	0.3	1.0	–8
		93 ± 8^c^		5^c^	8^c^			
		83 ± 11^d^		7^d^	13^d^			
retrorsine *N*-oxide	0.996	94 ± 7^b^	96 ± 6	4^b^	7^b^	0.3	1.0	2
		102 ± 8^c^		5^c^	8^c^			
		91 ± 6^d^		6^d^	8^d^			
seneciphylline	0.999	78 ± 7^b^	85 ± 8	7^b^	9^b^	0.2	0.6	–39
		93 ± 4^c^		5^c^	5^c^			
		83 ± 7^d^		8^d^	8^d^			
heliotrine	0.996	82 ± 11^b^	84 ± 11	7^b^	13^b^	0.3	1.0	28
		74 ± 9^c^		11^c^	12^c^			
		95 ± 5^d^		5^d^	15^d^			
seneciphylline *N*-oxide	0.998	89 ± 9^b^	86 ± 5	4^b^	12^b^	0.3	1.0	24
		89 ± 6^c^		6^c^	7^c^			
		80 ± 4^d^		5^d^	10^d^			
heliotrine *N*-oxide	0.995	76 ± 7^b^	83 ± 13	9^b^	10^b^	0.3	1.0	–9
		98 ± 5^c^		5^c^	7^c^			
		74 ± 3^d^		4^d^	7^d^			
senecivernine	0.998	91 ± 10^b^	83 ± 9	11^b^	12^b^	0.2	0.7	11
		84 ± 9^c^		4^c^	11^c^			
		74 ± 5^d^		7^d^	8^d^			
senecionine	0.998	98 ± 8^b^	84 ± 12	8^b^	9^b^	0.2	0.7	47
		80 ± 4^c^		4^c^	4^c^			
		75 ± 7^d^		9^d^	10^d^			
senecivernine *N*-oxide	0.998	86 ± 9^b^	82 ± 7	7^b^	10^b^	0.3	1.0	11
		74 ± 5^c^		7^c^	9^c^			
		85 ± 9^d^		11^d^	12^d^			
senecionine *N*-oxide	0.999	88 ± 12^b^	87 ± 3	6^b^	13^b^	0.3	1.0	–35
		89 ± 9^c^		7^c^	11^c^			
		83 ± 3^d^		4^d^	14^d^			
echimidine	0.997	76 ± 10^b^	86 ± 12	6^b^	13^b^	0.2	0.7	43
		82 ± 8^c^		5^c^	9^c^			
		99 ± 6^d^		9^d^	10^d^			
echimidine *N*-oxide	0.999	89 ± 9^b^	88 ± 2	8^b^	10^b^	0.2	0.7	–25
		86 ± 9^c^		10^c^	11^c^			
		90 ± 8^d^		7^d^	8^d^			
senkirkin	0.997	88 ± 10^b^	86 ± 2	9^b^	11^b^	0.3	1.0	33
		84 ± 9^c^		7^c^	11^c^			
		85 ± 10^d^		7^d^	11^d^			
lasiocarpine	0.996	89 ± 11^b^	84 ± 7	9^b^	12^b^	0.3	1.0	3
		87 ± 7^c^		7^c^	9^c^			
		76 ± 8^d^		8^d^	10^d^			
lasiocarpine *N*-oxide	0.995	93 ± 9^b^	85 ± 9	10^b^	10^b^	0.3	1.0	–60
		75 ± 9^c^		7^c^	12^c^			
		88 ± 11^d^		11^d^	12^d^			

a Recovery: mean recovery obtained from nine samples (*n* = 9) spiked with the analytes at a known concentration level, and subjected to the proposed extraction procedure; intraday precision: six replicate extracts (*n* = 6) analyzed on the same day of an infusion sample spiked with the analytes at a known concentration level; Interday precision: three replicates extracts of a sample analyzed throughout three different days (*n* = 9) and spiked with the analytes at a known concentration level; MDL: method detection limit; MQL: method quantification limit; ME: matrix effect.

b Low spiked level (1 μg/L of flower infusion).

c Medium spiked level (5 μg/L).

d High spiked level (50 μg/L).

**Table 4:** Validation Parameters of the μSPEed® Procedure Proposed for the Determination of the Target PAs/PANOs in Hibiscus Infusion Samples^a^

analytes	linearity (*R*^2^)	accuracy		precision		MDL (μg/L)	MQL (μg/L)	ME (%)
		recovery	mean recovery	intraday	interday			
		(% ± SD)	(% ± SD)	(RSD%)	(RSD%)			
intermedine	0.993	82 ± 7^b^	88 ± 6	14^b^	17^b^	0.3	1.0	–24
		92 ± 7^c^		8^c^	9^c^			
		91 ± 5^d^		5^d^	5^d^			
lycopsamine	0.999	85 ± 10^b^	86 ± 12	7^b^	13^b^	0.3	1.0	–38
		98 ± 12^c^		4^c^	12^c^			
		75 ± 3^d^		4^d^	12^d^			
europine	0.995	91 ± 11^b^	86 ± 7	11^b^	12^b^	0.3	0.8	–22
		90 ± 10^c^		6^c^	11^c^			
		78 ± 6^d^		8^d^	9^d^			
europine *N*-oxide	0.994	101 ± 9^b^	91 ± 16	9^b^	13^b^	0.3	1.0	–17
		100 ± 9^c^		6^c^	9^c^			
		73 ± 5^d^		7^d^	9^d^			
intermedine *N*-oxide	0.998	95 ± 7^b^	82 ± 12	7^b^	12^b^	0.3	1.0	–22
		81 ± 11^c^		9^c^	14^c^			
		71 ± 6^d^		7^d^	8^d^			
lycopsamine *N*-oxide	0.995	89 ± 12^b^	89 ± 10	5^b^	13^b^	0.3	1.0	52
		99 ± 4^c^		3^c^	4^c^			
		80 ± 9^d^		8^d^	12^d^			
retrorsine	0.999	92 ± 8^b^	91 ± 8	5^b^	8^b^	0.3	1.0	–37
		98 ± 4^c^		4^c^	4^c^			
		82 ± 5^d^		6^d^	7^d^			
retrorsine *N*-oxide	0.994	101 ± 7^b^	95 ± 7	1^b^	7^b^	0.3	1.0	–21
		98 ± 4^c^		4^c^	5^c^			
		87 ± 5^d^		4^d^	6^d^			
seneciphylline	0.999	92 ± 12^b^	93 ± 7	4^b^	13^b^	0.2	0.8	–61
		87 ± 5^c^		5^c^	6^c^			
		100 ± 2^d^		2^d^	7^d^			
heliotrine	0.995	80 ± 11^b^	79 ± 4	9^b^	13^b^	0.2	0.7	–43
		74 ± 9^c^		12^c^	12^c^			
		82 ± 3^d^		4^d^	6^d^			
seneciphylline *N*-oxide	0.998	94 ± 11^b^	88 ± 7	7^b^	11^b^	0.3	1.0	29
		81 ± 9^c^		3^c^	11^c^			
		89 ± 4^d^		1^d^	5^d^			
heliotrine *N*-oxide	0.995	87 ± 4^b^	85 ± 4	4^b^	11^b^	0.3	1.0	–3
		80 ± 5^c^		2^c^	7^c^			
		87 ± 10^d^		8^d^	12^d^			
senecivernine	0.997	88 ± 10^b^	86 ± 9	11^b^	12^b^	0.3	1.0	–40
		77 ± 6^c^		8^c^	9^c^			
		94 ± 7^d^		8^d^	9^d^			
senecionine	0.999	82 ± 9^b^	84 ± 3	7^b^	11^b^	0.3	1.0	–20
		83 ± 11^c^		4^c^	13^c^			
		88 ± 8^d^		9^d^	10^d^			
senecivernine *N*-oxide	0.999	90 ± 2^b^	94 ± 6	3^b^	6^b^	0.3	1.0	–1
		92 ± 7^c^		7^c^	8^c^			
		101 ± 6^d^		6^d^	10^d^			
senecionine *N*-oxide	0.997	88 ± 11^b^	97 ± 10	12^b^	13^b^	0.3	1.0	51
		95 ± 7^c^		6^c^	8^c^			
		107 ± 2^d^		2^d^	8^d^			
echimidine	0.994	96 ± 7^b^	89 ± 12	7^b^	7^b^	0.1	0.3	40
		76 ± 3^c^		4^c^	7^c^			
		96 ± 5^d^		5^d^	5^d^			
echimidine *N*-oxide	0.998	99 ± 7^b^	95 ± 6	7^b^	8^b^	0.3	1.0	22
		98 ± 6^c^		6^c^	6^c^			
		88 ± 9^d^		4^d^	10^d^			
senkirkin	0.995	99 ± 7^b^	88 ± 10	7^b^	7^b^	0.2	0.7	26
		79 ± 12^c^		7^c^	15^c^			
		88 ± 9^d^		3^d^	10^d^			
lasiocarpine	0.997	94 ± 7^b^	91 ± 4	6^b^	7^b^	0.3	1.0	–29
		91 ± 7^c^		7^c^	8^c^			
		87 ± 10^d^		11^d^	12^d^			
lasiocarpine *N*-oxide	0.996	100 ± 9^b^	94 ± 7	4^b^	9^b^	0.3	0.9	–74
		96 ± 7^c^		3^c^	8^c^			
		86 ± 6^d^		3^d^	7^d^			

a Recovery: mean recovery obtained from nine samples (*n* = 9) spiked with the analytes at a known concentration level, and subjected to the proposed extraction procedure; intraday precision: six replicate extracts (*n* = 6) analyzed on the same day of an infusion sample spiked with the analytes at a known concentration level; interday precision: three replicates extracts of a sample analyzed throughout three different days (*n* = 9) and spiked with the analytes at a known concentration level; MDL: method detection limit; MQL: method quantification limit; ME: matrix effect.

b Low spiked level (1 μg/L of flower infusion).

c Medium spiked level (5 μg/L).

d High spiked level (50 μg/L).

On the other hand, the results obtained from the slopes of the matrix-matched and solvent-based standard calibration curves revealed the presence of ME, being more intense in the mallow matrix and less relevant in calendula, with the following trend: malva > hibiscus > calendula) (Figure S11). In mallow, 15 compounds were out of the ME range ±20% (Table 2 and Figure S11). As previously mentioned, values between −50% < MEs < −20% and 50% > MEs > 20% could be considered as a soft effect.^37,38^ However, in the case of mallow, 8 compounds still showed strong signal suppression with ME values between −53 and −77% (Table 2 and Figure S11). In the case of the hibiscus matrix, 17 compounds were out of the ME range ±20%, but in this case, six of them showed signal increase (lycopsamine *N*-oxide, seneciphylline *N*-oxide, senecionine *N*-oxide, echimidine, echimidine *N*-oxide, and senkirkin), whereas only two presented strong signal suppression (lasiocarpine *N*-oxide and seneciphylline) (Table 4 and Figure S11). On the other hand, although 13 compounds were out of the ME range ±20% in the calendula sample, this was the matrix less affected by the matrix interferences, as signal increase was observed for 7 analytes and only lasiocarpine *N*-oxide showed strong signal suppression (Table 3 and Figure S11). Although some analytes did not show ME, as most of them were affected by interferences in the three flower matrices, to reach a compromise, matrix-matched calibration curves were required for quantification, so that the errors associated to matrix suppression or matrix enhancement can be considered and compensated in calibration.

Method selectivity was successfully fulfilled. No interfering peaks were observed at the retention time of the analytes, which was in all cases within the interval ±2.5%. Moreover, the variations of the spectra obtained in the standard solutions and in the sample did not exceed ±30%. The method also showed good sensitivity. The MDLs estimated from the extracted ion chromatograms (Figures S3–S5) ranged from 0.2 to 0.3 μg/L for mallow and calendula and 0.1 to 0.3 μg/L for hibiscus, whereas MQLs were in the range 0.5–1.0 μg/L for mallow and calendula and 0.3–1.0 μg/L for hibiscus (Tables 2^3^,4).

Satisfactory results were also found for accuracy in all the concentration levels evaluated for all the analytes in the three matrices (Tables 2^3^,4), as the recovery values obtained in the three levels were within the range 70–120% as specified in the validation guidelines.^35,36^ In this sense, the overall average recovery values were in the range 79–91, 82–97, and 79–97% in the mallow, calendula, and hibiscus matrices, respectively (Tables 2^3^,4). Likewise, method precision assessed in terms of intraday repeatability and interday reproducibility was also satisfactory at the three validation levels tested in the three matrices, as RSD values for the target analytes were in all cases ≤20% (Tables 2^3^,4) as specified in the validation guidelines.^35,36^ For intraday precision, the RSD values obtained were lower than 14% and for interday precision the results were lower than 17% (Tables 2^3^,4).

Overall, as the analytical parameters tested fully accomplished the validation guidelines,^35,36^ the good analytical performance of the μSPEed procedure developed was demonstrated, so it can be reliably applied to the analysis of PAs and PANOs in herbal infusions samples. As can be observed in Table 1, the analytical parameters of the μSPEed method proposed are similar to those of the previously published methods for the analysis of PAs in teas and herbal tea samples. Therefore, μSPEed constitutes a reliable, quick, and sustainable alternative to previous existing methods, showing that it is possible to achieve good analytical performance with a miniaturized strategy.

### 3.3 Application of the μSPEed Procedure to the Analysis of Flower Infusions

**Figure 4:**
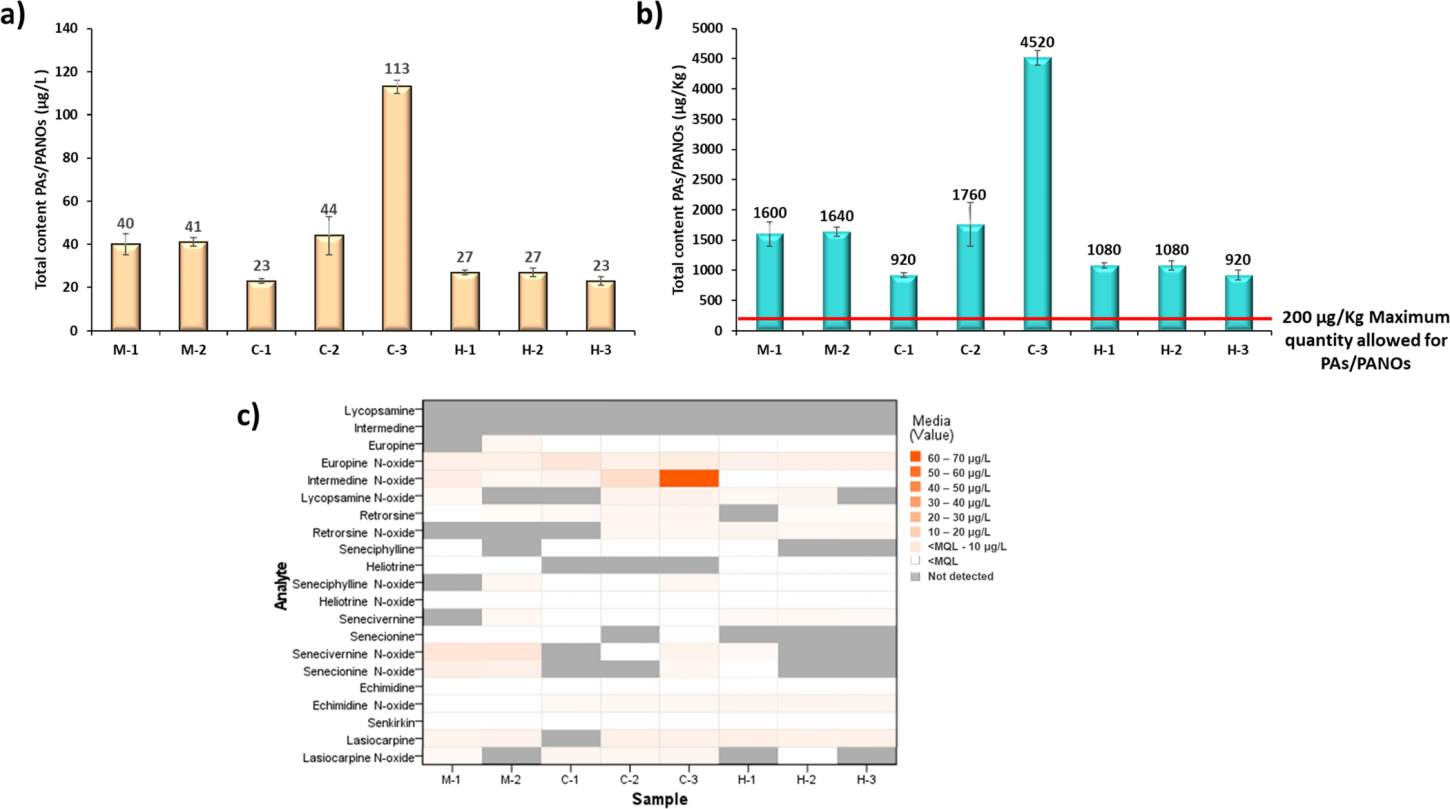
(a) Total content of pyrrolizidine alkaloids in infusion samples (μg/L), (b) total content of pyrrolizidine alkaloids in the dry plants (μg/kg) considering a transfer rate of 100%, and (c) heat map plot of the individual pyrrolizidine alkaloids found in the different edible flower infusion samples analyzed with the μSPEed method proposed. In the sample identification code, the first letter indicates the type of flower (M for mallow, C for calendula and H for hibiscus).

Once the μSPEed procedure developed was validated, it was then applied to the analysis of seven nonspiked infusion samples, including two mallow, three calendula, and three hibiscus infusion samples (Table S1), which were prepared and analyzed as previously explained in section 2.2. Matrix-matched calibration curves obtained in method validation were used for quantification. When the content found was below the MDL, it was considered as 0.0 μg/L (not detected), and contents between the MDL and MQL were indicated as <MQL (Table S2). Figure 4 shows the results obtained for the total content of PAs in the prepared infusion samples, an estimation of the total concentration of PAs in the dried flowers considering a 100% transfer rate of PAs and the individual analysis of PAs quantified in the samples analyzed.

As it can be observed, all the samples analyzed were contaminated with these alkaloids, but all the 21 PAs analyzed were not always present. In general, the hibiscus infusions were the least contaminated, whereas one of the calendula samples (C-3) showed the highest contamination value (113 μg/L) (Figure 4a,b). As stated in the introduction, to the best of our knowledge, there are no previous studies in the literature which analyze individual infusions of mallow and calendula for the determination of PAs. Regarding hibiscus, in the work of Picron et al., samples containing hibiscus are mentioned, but details are not provided, so it was assumed that these samples were referred to herbal mixed infusions with hibiscus in their composition.^12^ Only the work of Kwon et al. indicates the analysis of single hibiscus samples, but no PAs were detected in these samples.^11^ Therefore, for comparison purposes regarding the total content of PAs, Table 1 shows the range of the total PA concentration found in previous published works that performed determination of these alkaloids in other teas and herbal tea samples. As it is shown, the total content of PAs found in this work is within the range of other previous articles (Table 1).

Calendula belongs to the Asteraceae family, which it is known to be a PA-producing family.^40^ However, it was surprising to find important contamination levels of PAs in mallow and hibiscus infusions (40–41 and 23–27 μg/L, respectively) (Figure 4a,b), as these flowers belong to the Malvaceae family, which is known to be a non-PA-producing family. Nonetheless, currently, several contamination paths of PAs have been described (cross-contamination during harvest, horizontal natural transfer through soil, via bee pollination by collecting pollen and nectar from PA-producing plants, etc.),^2^ so it is possible to find these alkaloids in unexpected plant-based matrices. In fact, several authors have detected in previous published works high levels of PAs in teas and herbal infusions obtained from non-PA-producing plants, such as spearmint (Lamiaceae), melissa (Lamiaceae), green tea (Theaceae), lavender (Lamiaceae), and so on.^11,12,15,18,19,25^ For instance, Picron et al. detected a maximum level of 1936 μg/Kg in herbal teas without PA-producing plants in their ingredient list.^12^ Therefore, the concentrations levels found in this work for mallow and hibiscus (Figure 4a,b) are within the range reported by other authors for teas and infusions without PA-producing plants in their composition.^12,15,19,25^

Nonetheless, it is important to highlight that many of these works do not analyze infusion samples, but the dry product. Only a few of the works reviewed in Table 1 evaluated the levels of PAs in the infusion samples.^12,19,25,26,39^ This is important, because not always the transfer rate from the dry product to the infusion is 100%,^12,24−26^ so the actual concentration levels in the infusions can be different from the dry plant. For instance, Schulz et al. analyzed both the dry plant and their corresponding infusion samples, observing concentration ranges of PAs of 1127–5137 and 13–1080 μg/kg, respectively.^19^ Therefore, the analysis of the infusion samples provides a more reliable scenario of the real exposure of consumers to these contaminants. Moreover, the analysis of dry samples involves more complex sample treatment and higher consumption of reagents than the analysis of the liquid infusions (Table 1).

On the other hand, it was observed that the contamination of the infusions was mainly due to the *N*-oxide forms (PANOs) in the three types of flowers, which were more predominant than their corresponding PAs (Figure 4c and Table S2). This trend was also observed by other authors in previous studies for other infusion and tea samples.^12,16,19,25^

Regarding the individual analysis of the different flower matrices analyzed, the mallow samples presented similar total PAs values among them (40 and 41 μg/L) (Figure 4a,b), and they also showed more or less the same contamination profile, with a predominance of senecionine-type PAs contamination in this type of plants, mainly senecivernine *N*-oxide and senecionine *N*-oxide (Figure 4c and Table S2). These results suggest a possible cross-contamination of mallow with plant species belonging to the Asteraceae family, such as *Senecio vulgaris*.^12,41,42^ Nonetheless, although in a lesser extent, it is also worthy to highlight the presence of other PAs, such europine *N*-oxide, lasiocarpine (both heliotrine-type PAs), and intermedine *N*-oxide (lycopsamine-type PAs) in the mallow samples (Figure 4c and Table S2).

Likewise, as in the case of mallow, the hibiscus samples also presented similar total PAs values (23–27 μg/L) and contamination profile among them (Figure 4). However, in this case, the contamination of these flowers was mainly due to heliotrine-type PAs (europine *N*-oxide and lasiocarpine), followed by lycopsamine-type PAs (mainly echimidine *N*-oxide). Instead, less senecionine-type PA contamination was observed in the hibiscus samples (Figure 4c and Table S2). This may suggest that hibiscus flowers may present cross-contamination with *Borago spp.*, as according to previous studies, the contamination of heliotrine-type PAs and the occurrence of echimidine and its *N*-oxide are often associated to this plant.^42,43^

In contrast, the calendula samples were the ones which showed more variations among them, both in their total PA content and in their contamination profile (Figure 4). In this sense, sample C-1 was the least contaminated (23 μg/L), highlighting the occurrence of europine *N*-oxide (heliotrine-type PAs) on it (Figure 4 and Table S2). In contrast, a greater predominance of lycopsamine-type PAs was observed in samples C-2 and C-3, highlighting their content in intermedine *N*-oxide, especially in the case of C-3 (Figure 4c and Table S2). Moreover, it was observed that in C-3, the occurrence of senecionine-type PAs was greater than in the other calendula samples analyzed. In addition, also heliotrine-type PAs (europine *N*-oxide, lasiocarpine, and lasiocarpine *N*-oxide) were quantified in this sample (Figure 4c and Table S2). This PA profile found in the calendula samples matches with the PAs expected in this flower as previously reported for it,^40^ mainly in the case of sample C-3. This suggests the natural occurrence of PAs in this flower for being an Asteraceae plant, which also justifies the greater amount of PAs found in this sample in contrast with the other flowers analyzed in this work (mallow and calendula).

On the other hand, the total PA levels found in the different flower samples analyzed clearly exceeded in all cases the maximum levels established in the legislation for them (Figure 4b), 200 μg/kg as previously indicated.^27^ This is an important issue, as many people consume these products daily with therapeutic and dietary purposes due to their gastrointestinal, relaxing, anti-inflammatory, and expectorant effects, among others.^7−10^ In this sense, the safe daily intake of PAs established by the European Food Safety Authority (EFSA) is 0.007 μg per kg body weight.^1^ Accordingly, for a person of 60 kg of weight, the tolerable safe daily intake of PAs would be 0.42 μg. Therefore, considering the levels of total PAs found in the flower infusions analyzed (23–113 μg/L), the risk estimation of the intake of a daily cup (200 mL) of these infusions would be more than 10 and 55 times higher than the tolerable maximum daily dose established (4.6 and 22.6 μg/day, respectively). Therefore, the results obtained revealed the importance to monitor the occurrence of these alkaloids in any plant-derived product, highlighting the importance to control all types of plant-based products used for infusions, not only those that may naturally contain PAs, because as it has been shown in this study, as well as in other published works (Table 1), this type of products can be highly contaminated with these alkaloids entailing a concerning health risk for consumers.

Overall, it is important to develop quick, selective, and sensitive analytical procedures that contribute to improve the quality control of teas and herbal infusions and ensure food safety in a sustainable way, such as the one developed in this work. In this work, a quick and sustainable analytical methodology based on extraction by μSPEed technique combined with UHPLC-IT-MS/MS was successfully developed and validated to monitor the occurrence of PAs in different flower infusion samples. It involved minimal consumption of organic solvents and sample, providing high extraction efficiency in very short extraction time, being easier and more advantageous than other conventional extraction techniques, such as SPE and QuEChERS. Moreover, the μSPEed technique proved to be very suitable for the extraction of liquid samples, such as the case of teas and herbal infusions. This is very interesting for the determination of PAs in this type of samples, because it is more convenient to perform the analysis of infusions samples rather than the analysis of the dry plant, as not always the transfer rate of PAs from the dry product to the infusion is 100%. In this sense, the analysis of infusion samples allows a more reliable knowledge of the real exposure of consumers to these contaminants through the intake of these products. Additionally, the analysis of dry samples often involves more tedious sample treatment and higher amount of reagents is required. Moreover, the results obtained in this work highlighted the concerning high degree of PA contamination in plant-based products used for teas and infusions, which may entail a great risk to the health of consumers if they are consume continuously. Thus, this works represents and efficient approach to contribute and improve food safety and quality control of food items by the monitorization of PAs in a cost-effective and sustainable way.

## Supporting Information Available

Information of the edible dried flowers analyzed, edible flower infusions prepared and analyzed, retention time, precursor ion, and product ions of the analytes analyzed with the UHPLC-IT-MS/MS chromatographic method used, extracted ion chromatograms of pyrrolizidine alkaloids at 1 μg/L with their signal-to-noise ratio (S/N) analyzed from the spiked flower samples, recovery values obtained from the μSPEed analysis of the different flower infusions spiked with the analytes (50 μg/L of each analyte) using C18 cartridge with 5 or 3 extraction cycles in sample loading and considering the effect of the washing step, comparison of recovery values obtained from the μSPEed analysis of the flower infusions spiked with the analytes (50 μg/L of each analyte) using C18 cartridge with different extraction cycles (3 and 5 cycles) and without washing step, recovery values obtained from the μSPEed analysis of the flower infusions spiked with the analytes (50 μg/L of each analyte) using different elution volumes of methanol (100, 200, and 250 μL), 2D plot of the matrix effect of the analytes/retention time obtained in the different edible flower infusion samples with the μSPEed method proposed, and content of PAs/PANOs (μg/L) quantified in the different edible flower infusion samples analyzed by the μSPEed method proposed (PDF).
