# Supplementary material for: Quick and Green Microextraction of Pyrrolizidine Alkaloids from Infusions of Mallow, Calendula, and Hibiscus Flowers Using Ultrahigh-Performance Liquid Chromatography Coupled to Tandem Mass Spectrometry Analysis
Source: J Agric Food Chem. 2022 Jun 17;70(25):7826–41. doi: 10.1021/acs.jafc.2c02186 (PMC9930110; doi:10.1021/acs.jafc.2c02186)
Supplement: Supplementary file 1 — jf2c02186_si_001.pdf [file jf2c02186_si_001.pdf]

Supplementary material

Quick and green microextraction of pyrrolizidine alkaloids from infusions of mallow, calendula and hibiscus flowers using ultrahigh-performance liquid chromatography coupled to tandem mass spectrometry analysis

Natalia Casado, Begoña Fernández-Pintor, Sonia Morante-Zarcero, Isabel Sierra *

Departamento de Tecnología Química y Ambiental, E.S.C.E.T, Universidad Rey Juan Carlos, C/ Tulipán s/n, 28933 Móstoles, Madrid, Spain

* Corresponding author: Tel.: (+34) 914887018; fax: (+34) 914888143.

E-mail addresses: natalia.casado@urjc.es; begona.fernandez@urjc.es; sonia.morante@urjces; isabel.sierra@urjc.es

Table S1. Information of the edible dried flowers analyzed.

Sample	Product origin	Description
M-1	Spain	Bulk bag of dried mallow flowers (*Alcea rosea*, plant family Malvaceae*)*
M-2	Spain	Bulk bag of dried mallow flowers (*Alcea rosea*, plant family Malvaceae*)*
C-1	Spain	Bulk bag of dried calendula flowers (*Calendula officinalis*, plant family Asteraceae*)*
C-2	Spain	Bulk bag of dried calendula flowers (*Calendula officinalis*, plant family Asteraceae*)*
C-3	Portugal	Bulk bag of dried calendula flowers (*Calendula officinalis*, plant family Asteraceae*)*
H-1	Spain	Bulk bag of dried hibiscus flowers (*Hibiscus sabdariffa*, plant family Malvaceae*)*
H-2	Spain	Bulk bag of dried hibiscus flowers (*Hibiscus sabdariffa*, plant family Malvaceae*)*
H-3	Portugal	Bulk bag of dried hibiscus flowers (*Hibiscus sabdariffa*, plant family Malvaceae*)*

In the sample identification code, the first letter indicates de type of flower (M for mallow, C for calendula and H for hibiscus).

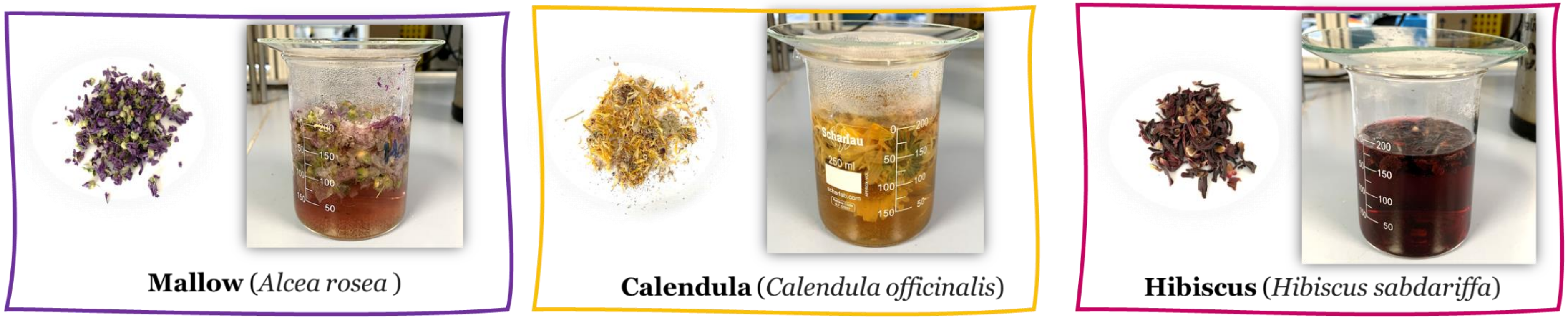

Fig. S1 Edible flower infusions prepared and analyzed.

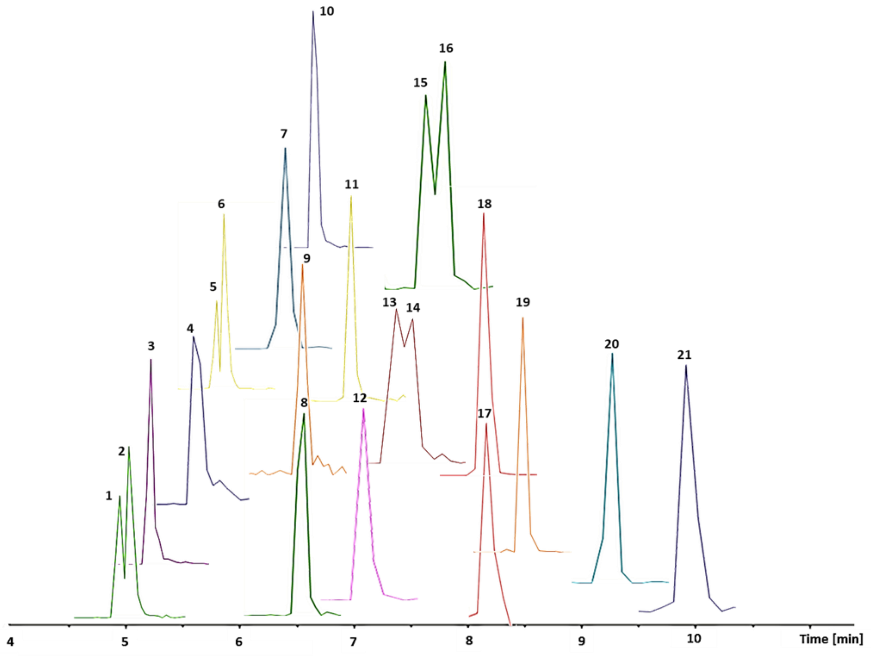

Analyte [Precursor ion (m/z)]	Retention time (min)	MS^2 Product ions (m/z)
1. Intermedine [299]	4.8	138*, 120
2. Lycopsamine [299]	5.1	138*, 120
3. Europine [329]	5.3	253*, 138
4. Europine N-oxide [345]	5.7	327*, 171.5
5. Intermedine N-oxide [315]	5.8	225, 171.5*
6. Lycopsamine N-oxide [315]	5.9	171.5*, 138
7. Retrorsine [351]	6.4	323*, 275
8. Retrorsine N-oxide [367]	6.6	339*, 245
9. Seneciphylline [333]	6.6	305*, 120
10. Heliotrine [313.5]	6.7	138*, 120
11. Seneciphylline N-oxide [350]	7.0	321*, 118
12. Heliotrine N-oxide [329]	7.1	171*, 136
13. Senecivernine [335]	7.4	307*, 120
14. Senecionine [335]	7.5	307*, 120
15. Senecivernine N-oxide [351]	7.6	323*, 219.5
16. Senecionine N-oxide [352]	7.8	220, 118*
17. Echimidine N-oxide [398]	8.2	220, 120*
18. Echimidine [413]	8.2	395*, 351
19. Senkirkin [365]	8.5	167.5*, 150
20. Lasiocarpine [411]	9.3	335*, 219.5
21. Lasiocarpine N-oxide [328]	9.9	409*, 352

** Ions used for quantification*

Fig. S2 Retention time, precursor ion and product ions of the analytes analyzed with the UHPLC-IT-MS/MS chromatographic method used.

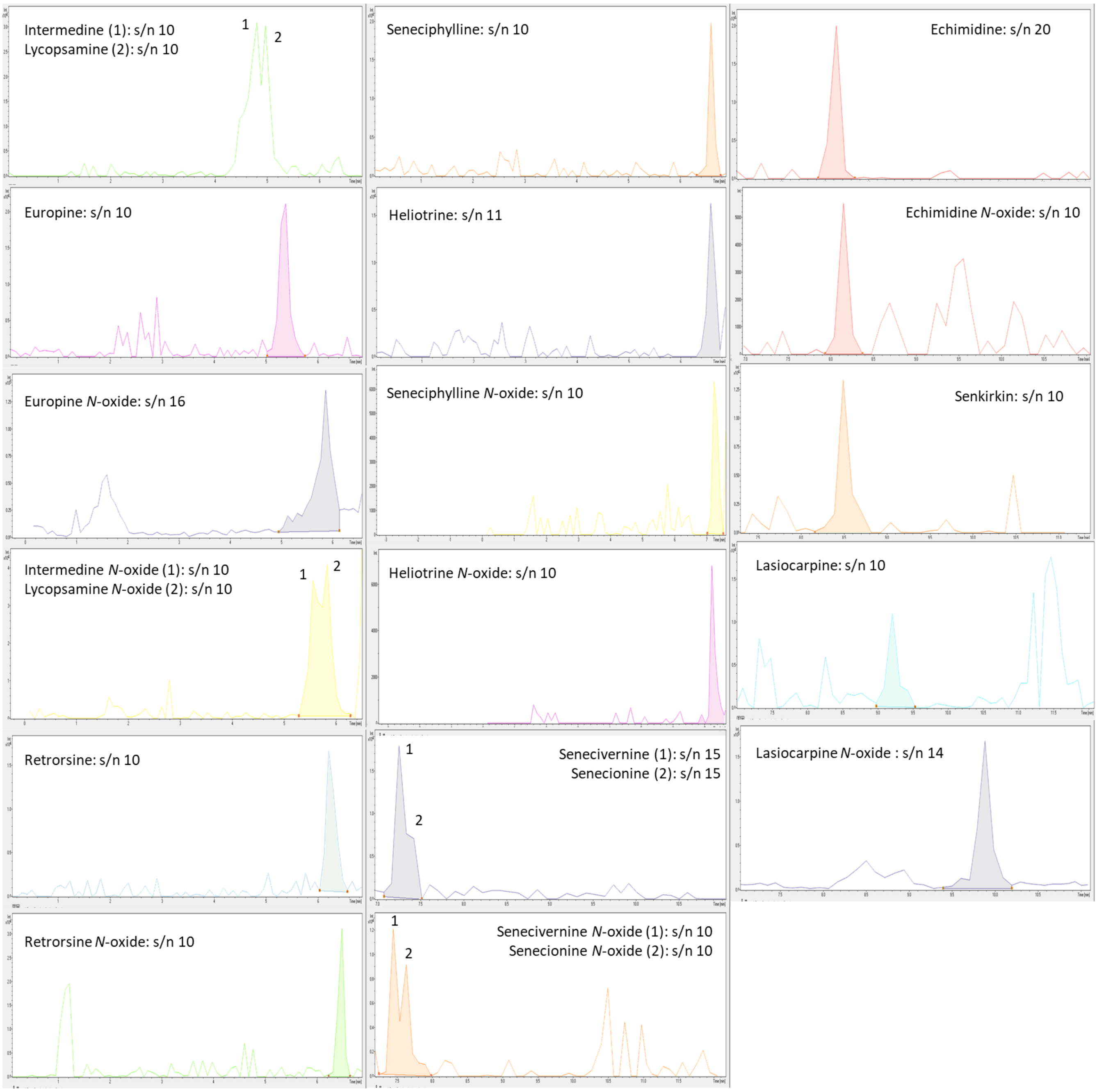

Fig. S3 Extracted ion chromatograms of pyrrolizidine alkaloids at 1 µg/L with their signal-to-noise ratio (S/N) analyzed from a spiked mallow sample.

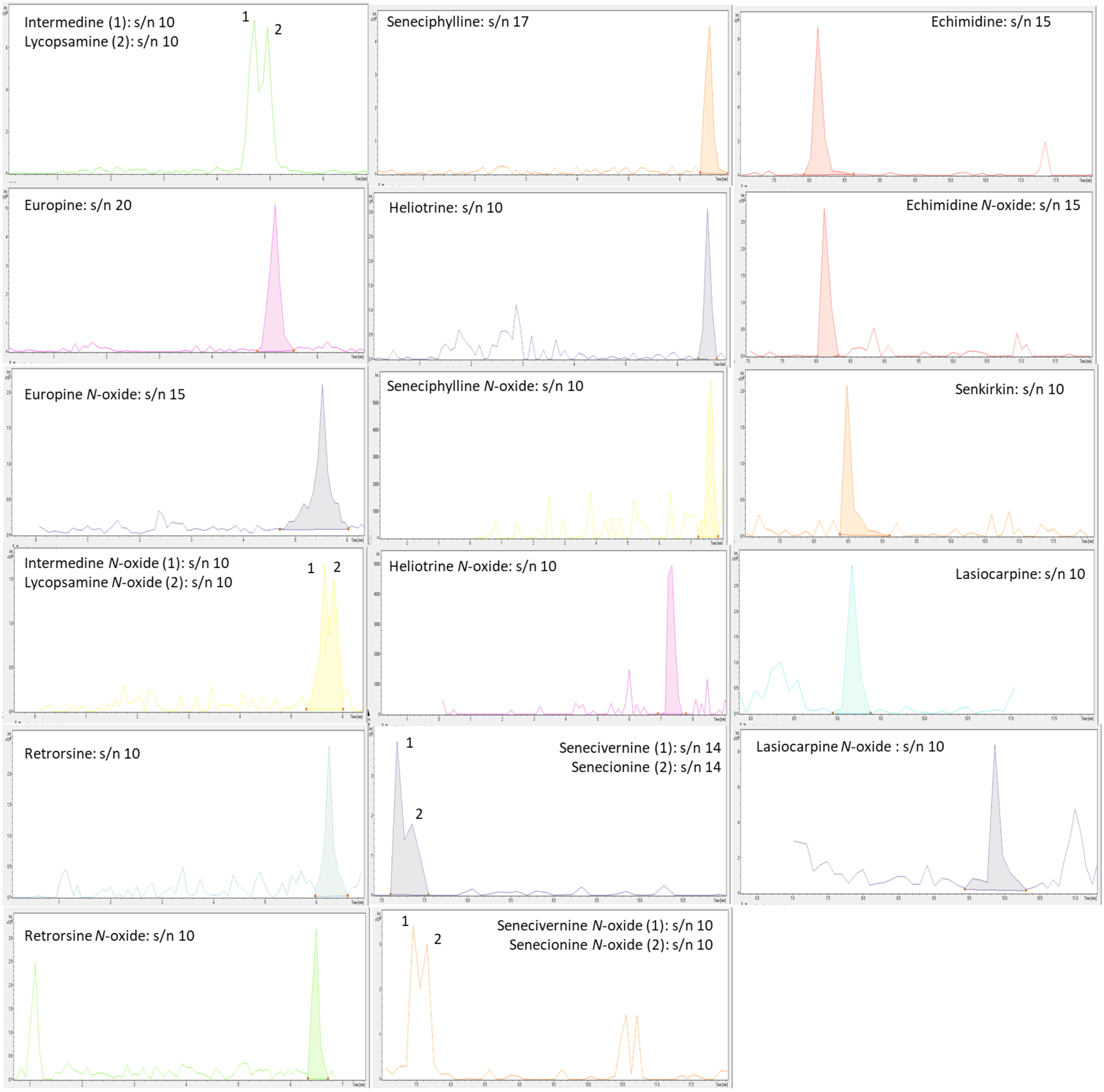

Fig. S4 Extracted ion chromatograms of pyrrolizidine alkaloids at 1 µg/L with their signal-to-noise ratio (S/N) analyzed from a spiked calendula sample.

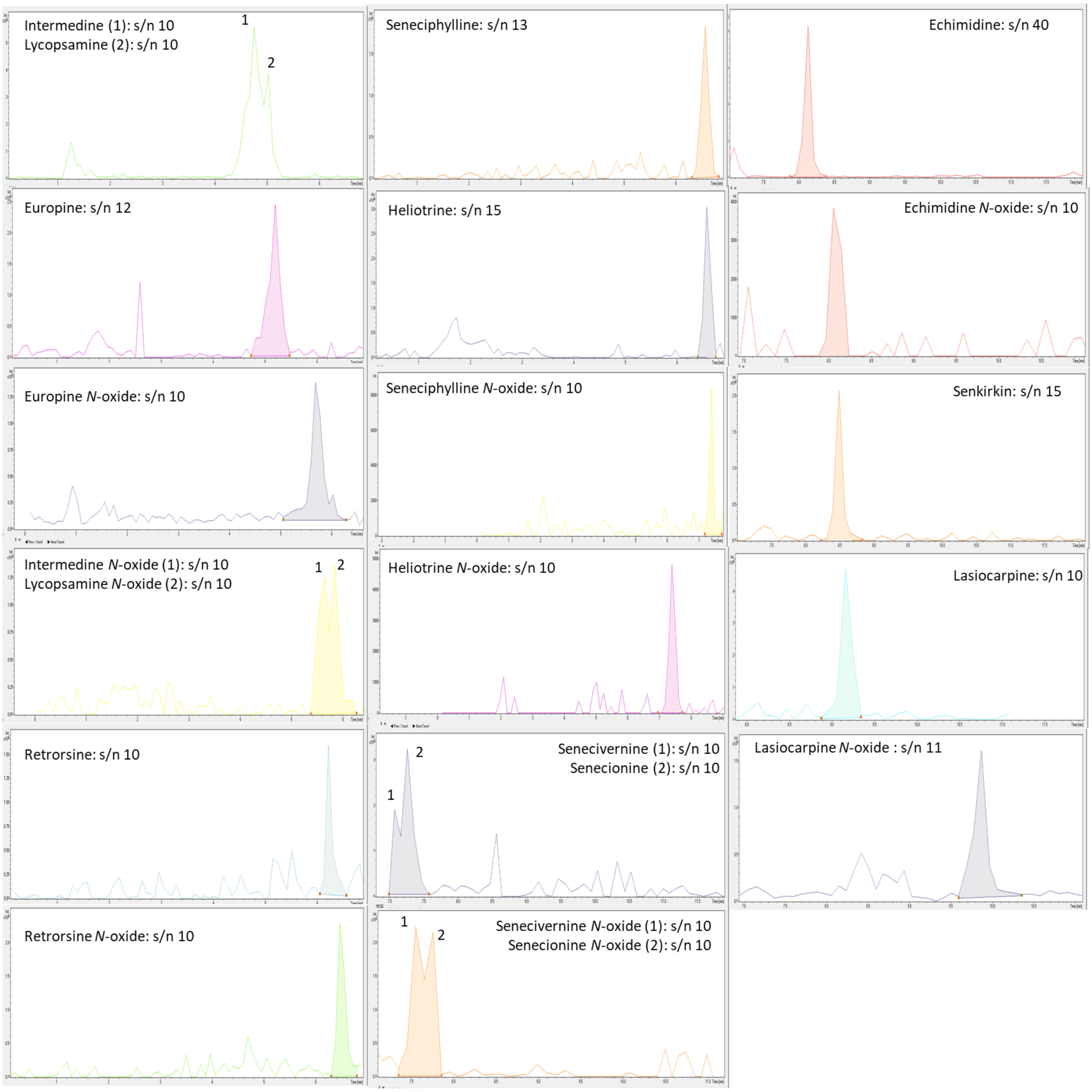

Fig. S5 Extracted ion chromatograms of pyrrolizidine alkaloids at 1 µg/L with their signal-to-noise ratio (S/N) analyzed from a spiked hibiscus sample.

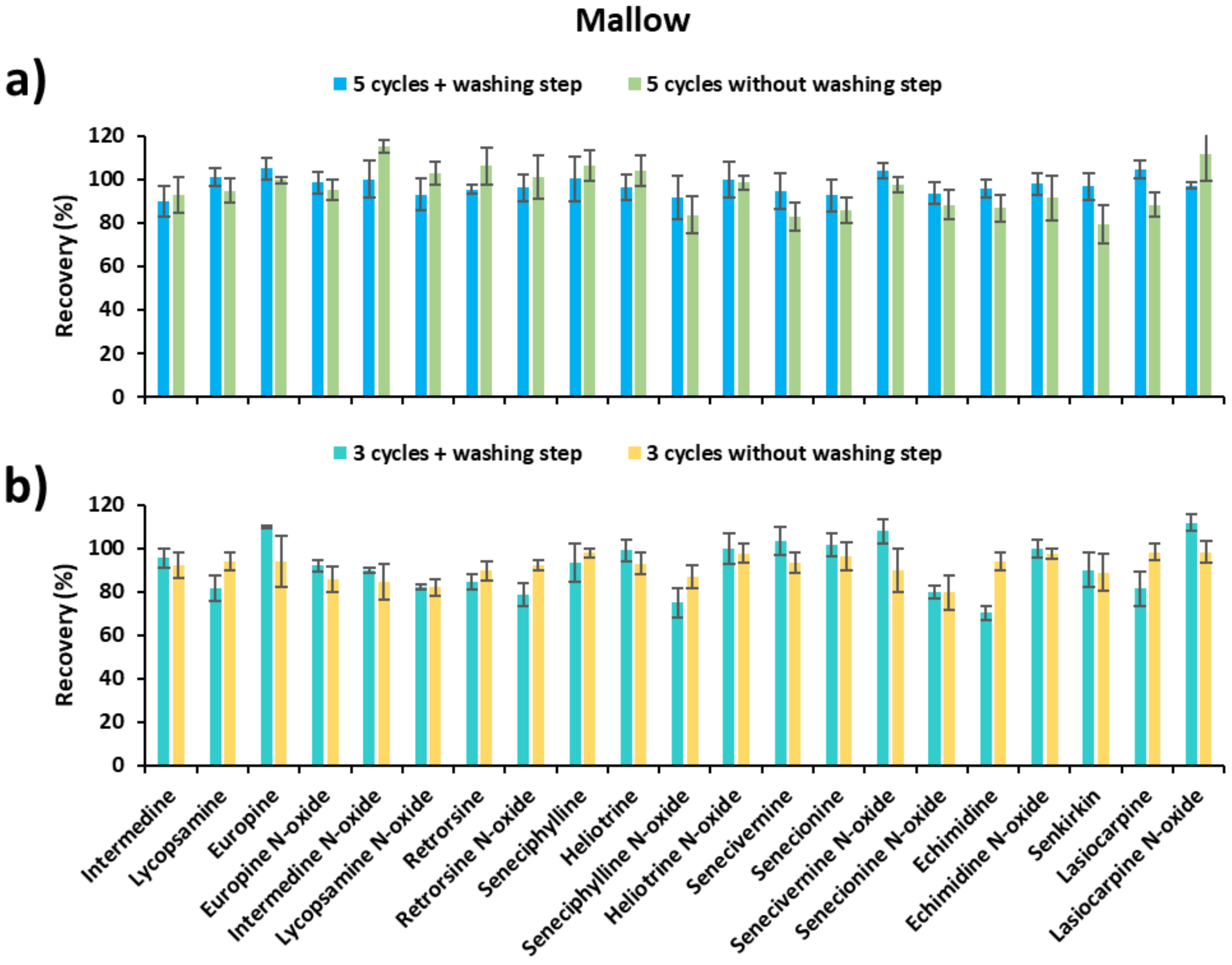

Fig. S6 Recovery values obtained from the µSPEed® analysis of mallow infusions spiked with the analytes (50 µg/L of each analyte) using C18 cartridge with (a) 5 extraction cycles and (b) 3 extraction cycles in sample loading and considering the effect of the washing step.

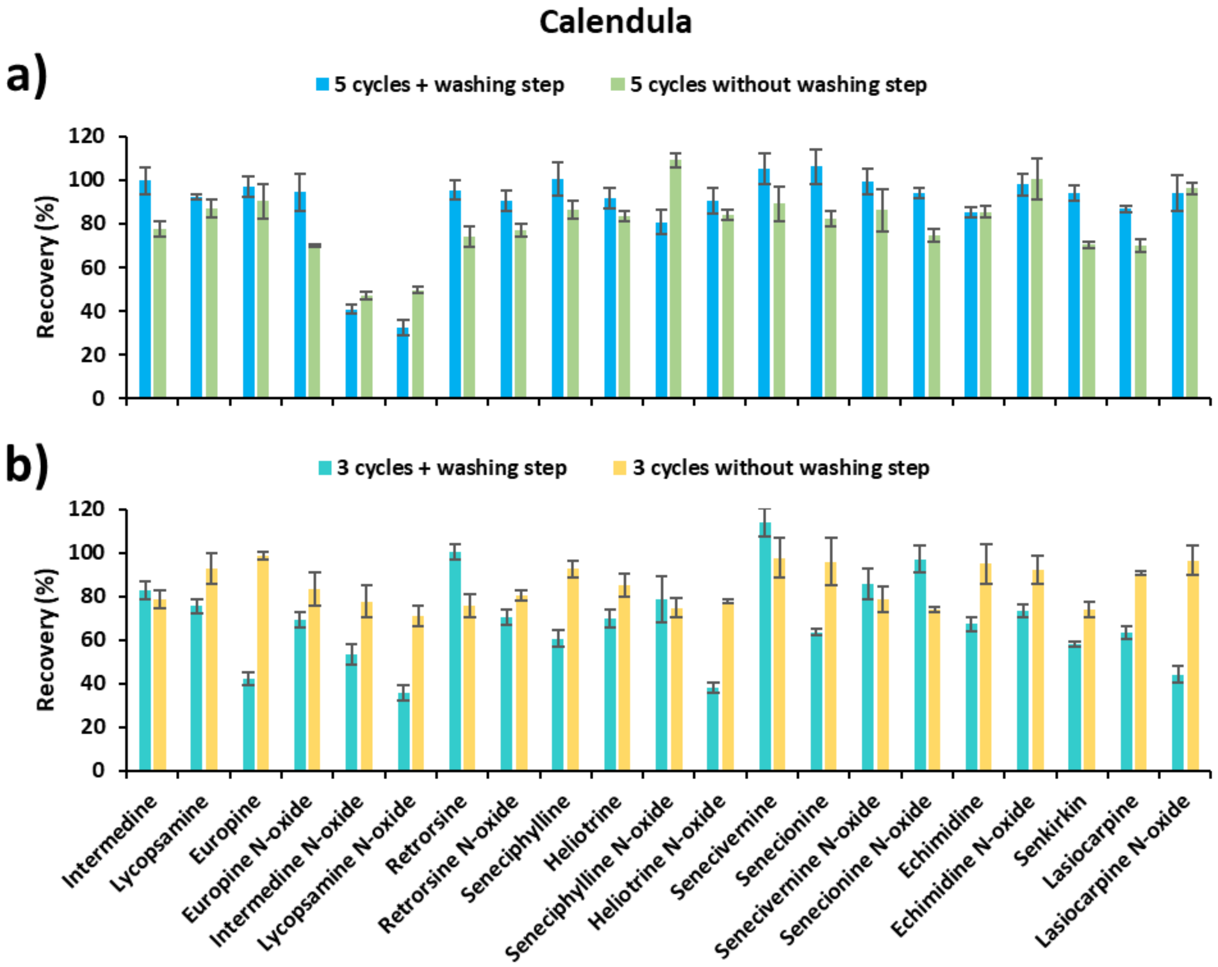

Fig. S7 Recovery values obtained from the µSPEed® analysis of calendula infusions spiked with the analytes (50 µg/L of each analyte) using C18 cartridge with (a) 5 extraction cycles and (b) 3 extraction cycles in sample loading and considering the effect of the washing step.

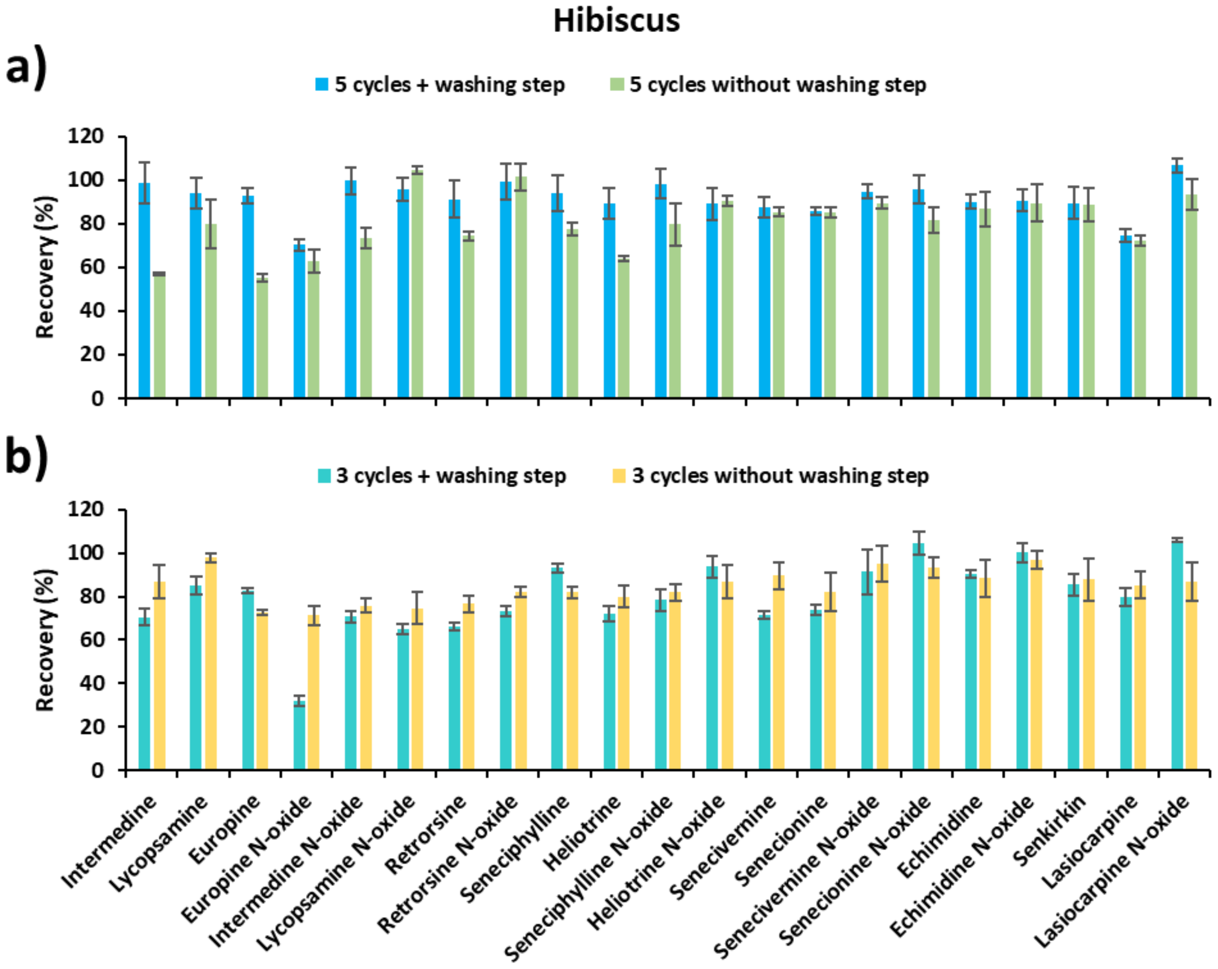

Fig. S8 Recovery values obtained from the µSPEed® analysis of hibiscus infusions spiked with the analytes (50 µg/L of each analyte) using C18 cartridge with (a) 5 extraction cycles and (b) 3 extraction cycles in sample loading and considering the effect of the washing step.

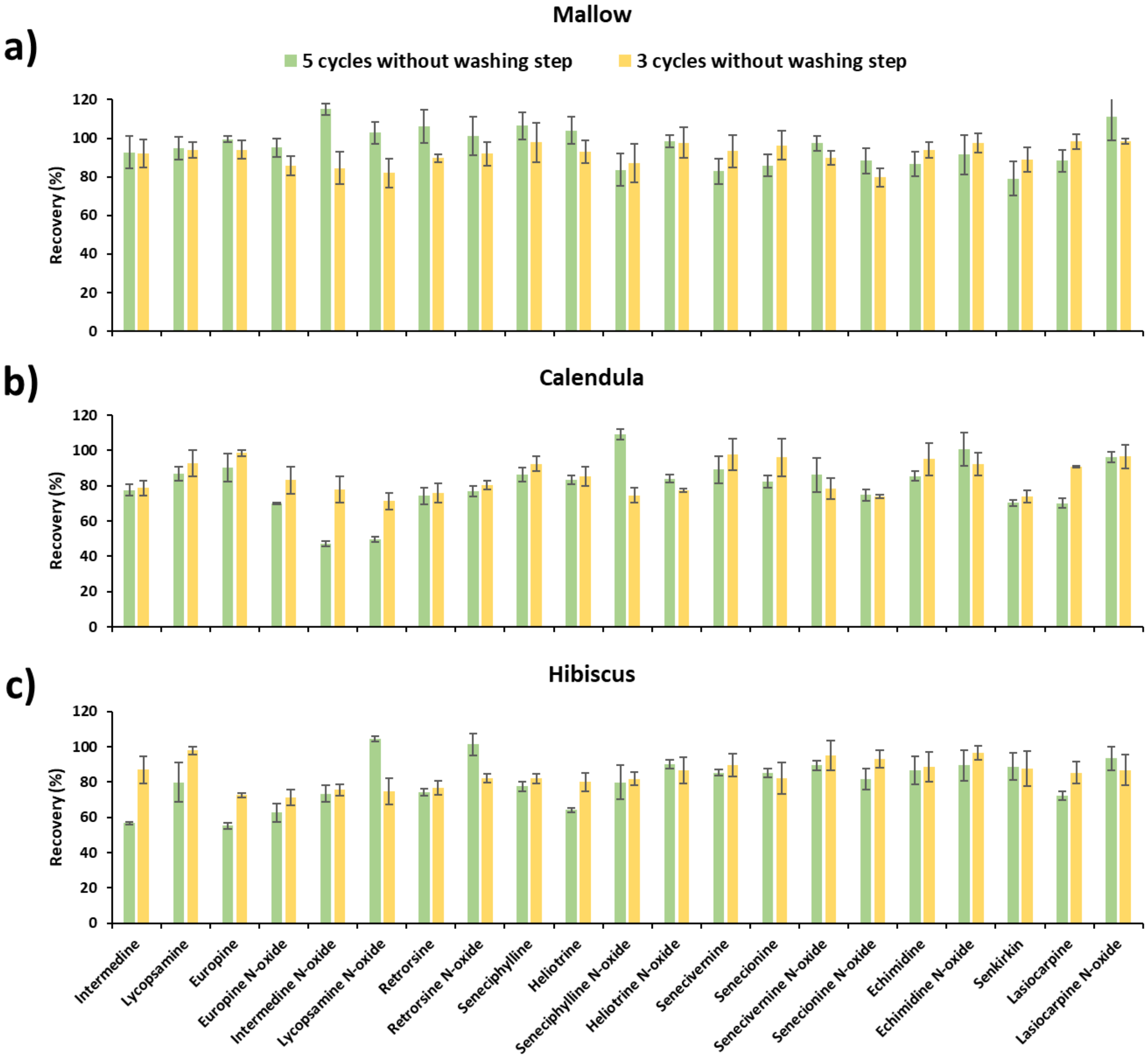

Fig. S9 Comparison of recovery values obtained from the µSPEed® analysis of (a) mallow, (b) calendula and (c) hibiscus infusions spiked with the analytes (50 µg/L of each analyte) using C18 cartridge with different extraction cycles (3 and 5 cycles) and without washing step.

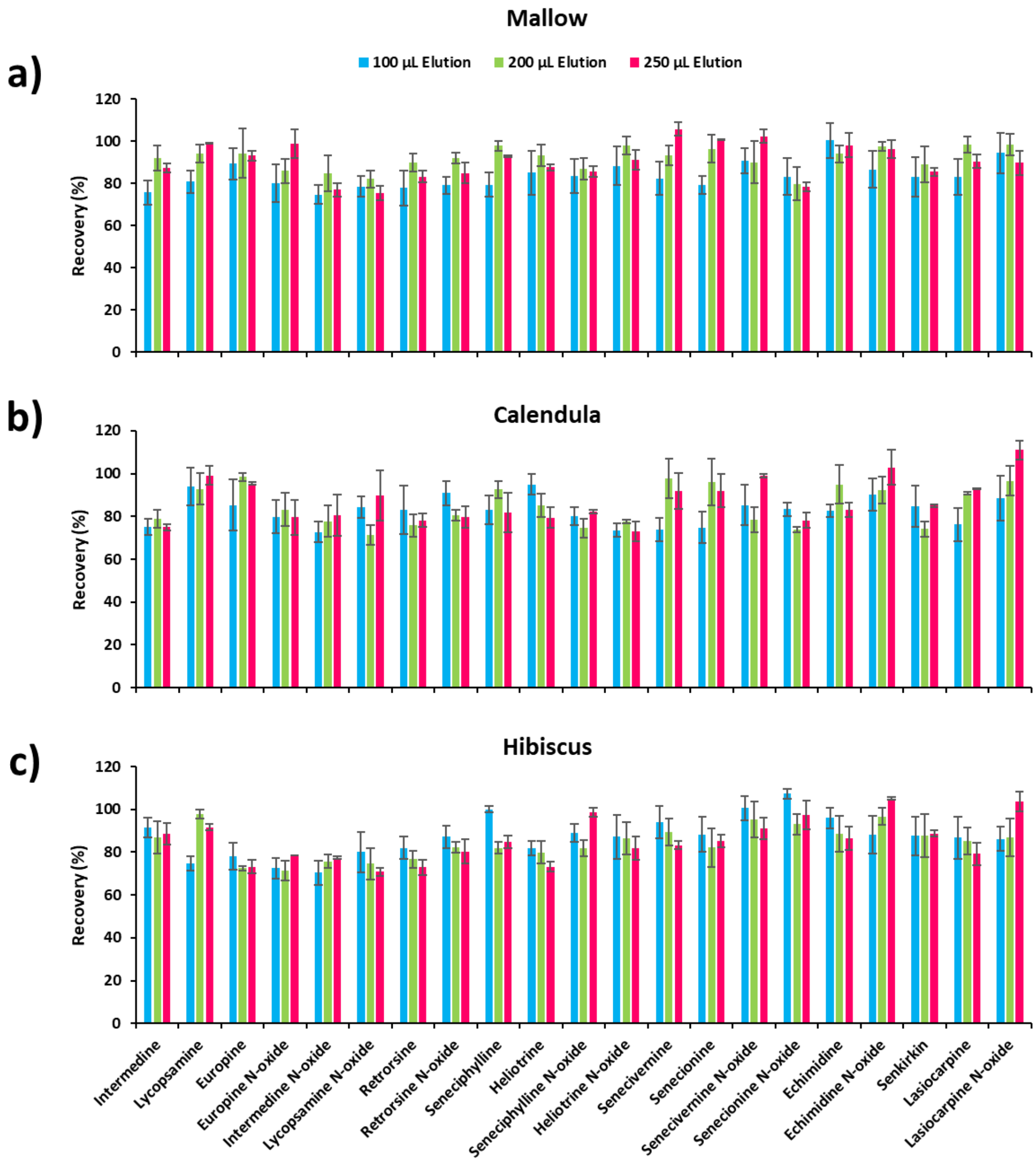

Fig. S10 Recovery values obtained from the µSPEed® analysis of **(a)** mallow, **(b)** calendula and **(c)** hibiscus infusions spiked with the analytes (50 µg/L of each analyte) using different elution volumes of methanol (100, 200 and 250 µL). Extraction conditions: C18 cartridge, cartridge conditioning with 2 x 100 µL methanol and 2 x 100 µL cycles; 3 x 100 µL sample loading and elution.

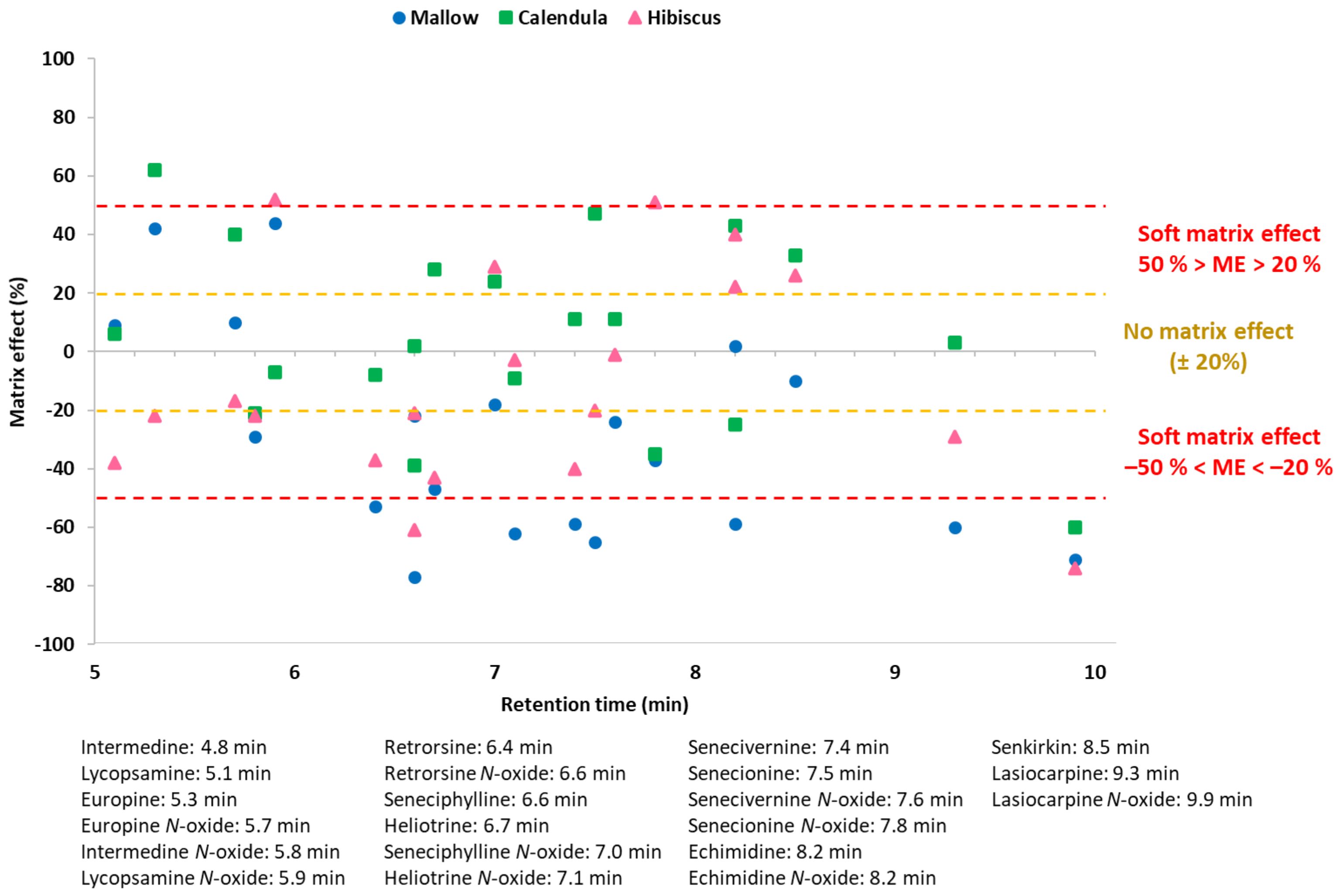

Fig. S11 2D plot of the matrix effect of the analytes/retention time obtained in the different edible flower infusion samples with the µSPEed® method proposed.

Table S2. Content of PAs/PANOs (µg/L) quantified in the different edible flower infusion samples analyzed by the µSPEed® method proposed.

	**M-1**	**M-2**	**C-1**	**C-2**	**C-3**	**H-1**	**H-2**	**H-3**
**Intermedine**	n.d.	n.d.	n.d.	n.d.	n.d.	n.d.	n.d.	n.d.
**Lycopsamine**	n.d.	n.d.	n.d.	n.d.	n.d.	n.d.	n.d.	n.d.
**Europine**	n.d.	3.50 ± 0.02	<MQL	<MQL	<MQL	<MQL	<MQL	<MQL
**Europine N-oxide**	6 ± 1	5.8 ± 0.2	10.0 ± 0.4	5.5 ± 0.7	7.5 ± 0.2	5.1 ± 0.2	6 ± 2	6 ± 2
**Intermedine N-oxide**	7 ± 5	3 ± 2	3.8 ± 0.3	14 ± 9	70 ± 3	<MQL	<MQL	<MQL
**Lycopsamine N-oxide**	2.7 ± 0.2	n.d.	n.d.	4.4 ± 0.4	4.9 ± 0.8	2.91 ± 0.01	4.48 ± 0.03	n.d.
**Retrorsine**	<MQL	1.4 ± 0.3	3.2 ± 0.1	3.5 ± 0.3	3.8 ± 0.4	n.d.	1.5 ± 0.3	1.3 ± 0.52
**Retrorsine N-oxide**	n.d.	n.d.	n.d.	3.5 ± 0.8	3.5 ± 0.4	4.5 ± 0.7	3.1 ± 0.5	3.5 ± 0.4
**Seneciphylline**	<MQL	n.d.	<MQL	<MQL	<MQL	<MQL	n.d	n.d.
**Heliotrine**	<MQL	<MQL	n.d.	n.d.	n.d.	<MQL	<MQL	<MQL
**Seneciphylline N-oxide**	n.d.	3.41 ± 0.08	<MQL	<MQL	3.4 ± 0.1	<MQL	<MQL	<MQL
**Heliotrine N-oxide**	<MQL	<MQL	<MQL	<MQL	<MQL	<MQL	<MQL	<MQL
**Senecivernine**	n.d.	2.38 ± 0.02	<MQL	<MQL	<MQL	2.2 ± 0.3	2.4 ± 0.3	2.19 ± 0.04
**Senecionine**	<MQL	<MQL	<MQL	n.d.	<MQL	n.d.	n.d.	n.d.
**Senecivernine N-oxide**	11.0 ± 0.7	10.7 ± 0.4	n.d.	<MQL	4.5 ± 0.8	2.23 ± 0.09	n.d.	n.d.
**Senecionine N-oxide**	7 ± 1	6 ± 1	n.d.	n.d.	3 ± 1	<MQL	n.d	n.d.
**Echimidine**	<MQL	<MQL	<MQL	<MQL	<MQL	<MQL	<MQL	<MQL
**Echimidine N-oxide**	<MQL	<MQL	2.6 ± 0.7	3.5 ± 0.4	3.3 ± 0.6	4.0 ± 0.1	4.2 ± 0.3	4.0 ± 0.3
**Senkirkin**	<MQL	<MQL	<MQL	<MQL	<MQL	<MQL	<MQL	<MQL
**Lasiocarpine**	4.4 ± 0.2	5.0 ± 0.4	n.d.	5.8 ± 0.6	5.4 ± 0.2	6 ± 1	5.4 ± 0.6	5.6 ± 0.3
**Lasiocarpine N-oxide**	2.1 ± 0.5	n.d.	3.8 ± 0.1	3.6 ± 0.1	3.8 ± 0.2	n.d.	<MQL	n.d.
**Total amount**	**40 ± 5**	**41 ± 2**	**23 ± 1**	**44 ± 9**	**113 ± 3**	**27 ± 1**	**27 ± 2**	**23 ± 2**

n.d. = not detected; <MQL: below the limit of quantification of the method. In the sample identification code, the first letter indicates the type of flower (M for mallow, C for calendula and H for hibiscus).
